# Mountaintop removal coal mining impacts on structural and functional indicators in Central Appalachian streams

**DOI:** 10.3389/frwa.2022.988061

**Published:** 2023-01-19

**Authors:** Roger A. Burke, Ken M. Fritz, Brent R. Johnson, Rachel Price

**Affiliations:** 1 United States Environmental Protection Agency (USEPA), Center for Environmental Measurement and Modeling (CEMM), Athens, GA, United States; 2 United States Environmental Protection Agency (USEPA), Center for Environmental Measurement and Modeling (CEMM), Cincinnati, OH, United States

**Keywords:** ecological integrity, structural indicators, functional indicators, coal mining, streams

## Abstract

Mountaintop removal coal mining (MTR) has been a major source of landscape change in the Central Appalachians of the United States (US). Changes in stream hydrology, channel geomorphology and water quality caused by MTR coal mining can lead to severe impairment of stream ecological integrity. The objective of the Clean Water Act (CWA) is to restore and maintain the ecological integrity of the Nation’s waters. Sensitive, readily measured indicators of ecosystem structure and function are needed for the assessment of stream ecological integrity. Most CWA assessments rely on structural indicators; inclusion of functional indicators could make these assessments more holistic and effective. The goals of this study were: (1) test the efficacy of selected carbon (C) and nitrogen (N) cycling and microbial structural and functional indicators for assessing MTR coal mining impacts on streams; (2) determine whether indicators respond to impacts in a predictable manner; and (3) determine if functional indicators are less likely to change than are structural indicators in response to stressors associated with MTR coal mining. The structural indicators are water quality and sediment organic matter concentrations, and the functional indicators relate to microbial activity and biofilm production. Seasonal measurements were conducted over the course of a year in streams draining small MTR-impacted and forested watersheds in the Twentymile Creek watershed of West Virginia (WV). Five of the eight structural parameters measured had significant responses, with all means greater in the MTR-impacted streams than in the forested streams. These responses resulted from changes in source or augmentation of the original source of the C and N structural parameters because of MTR coal mining. Nitrate concentration and the stable carbon isotopic ratio of dissolved inorganic carbon were the most effective indicators evaluated in this study. Only three of the fourteen functional indicators measured had significant responses to MTR coal mining, with all means greater in the forested streams than in the MTR-impacted streams. These results suggest that stressors associated with MTR coal mining caused reduction in some aspects of microbial cycling, but resource subsidies may have counterbalanced some of the inhibition leading to no observable change in most of the functional indicators. The detritus base, which is thought to confer functional stability, was likely sustained in the MTR-impacted streams by channel storage and/or leaf litter inputs from their largely intact riparian zones. Overall, our results largely support the hypothesis that certain functional processes are more resistant to stress induced change than structural properties but also suggest the difficulty of identifying suitable functional indicators for ecological integrity assessment.

## Introduction

A major goal of the 1990 amendment to the United States (US) Clean Air Act was to reduce acid precipitation. In response to this, coal production in West Virginia shifted from the high-sulfur coals in the north to the low-sulfur coals in the steep mountainous terrain of the south (Peng, 2000). Mountaintop removal coal mining (MTR) was a cost-effective technique that allowed the profitable recovery of coal seams in mountain ridges in the early twenty-first century (Peng, 2000). At the time of this study, the MTR process consisted of: (1) removing forest cover and soil; (2) blasting and removing overlying sedimentary rock layers (overburden or spoil); (3) mining the coal; (4) returning most of the overburden to the ridges; (5) dumping the excess overburden in adjacent valleys, which creates valley fills and buries stream channels; and (6) reclaiming the site (Griffith et al., 2012). The MTR process exposes minerals containing reduced sulfur and unweathered carbonates to air, resulting in the formation of sulfuric acid which dissolves the carbonates, releasing huge quantities of dissolved inorganic carbon (DIC), and calcium, magnesium, and sulfate ions to receiving streams which causes elevated conductivity (Ross et al., 2018). Reclamation entails site stabilization by compaction and terracing, reconstructing the soil column, constructing diversion channels to support hydrological stability, and rebuilding the landscape by restoring the vegetation (Feng et al., 2019). Largely because the MTR technique opened up such vast areas to profitable coal production, surface coal mining became the single greatest driver of land use change in the heavily forested Central Appalachians region of the US in recent decades (Drummond and Loveland, 2010). The cumulative area impacted by surface coal mining between 1976 and 2015 was estimated to be 5,900 km^2^, about 7.1% of Central Appalachia (Pericak et al., 2018). Using data from the U.S Energy Information Administration (U.S. Energy Information Administration, 2015, 2019) we calculated that the MTR technique accounted for 41% of the total surface coal production from 2008 to 2014 in West Virginia and Kentucky, which dominate MTR coal mining in Central Appalachia (Supplementary Table 1).

The objective of the US Clean Water Act (CWA) is to restore and maintain the ecological integrity of US waters. Human modifications of watershed land use often stress stream ecosystems and diminish their ecological integrity (Allan, 2004). Appropriate species, structures, and functions at multiple hierarchical levels are required for ecological integrity (Dale and Beyeler, 2001). Structures include the composition of the biological community, abiotic materials such as nutrients and other chemical components, and other features such as habitat structure, temperature, streamflow, and light, and functions include biological energy flows and nutrient cycling (Odum, 1962). The sediment microbial community plays a key role in several stream ecosystem functions including decomposition of organic matter and nutrient transformations (Findlay, 2010). These functions are controlled by several factors including the biodegradability of the organic matter undergoing decomposition, temperature, available electron acceptors, and a suitable microbial community (Jones, 1985). The energy yields of the various oxidation-reduction (redox) reactions associated with organic matter decomposition when denitrification occurs favor the use of electron acceptors in the following order: oxygen, nitrate, manganese (Mn IV), iron (Fe III), sulfate, and bicarbonate (Bethke et al., 2011). Assessing the impacts of MTR coal mining on the ecological integrity of Central Appalachian streams should consider structural and functional features of organic matter and nutrient cycling.

Tools for assessing ecological integrity are divided into two categories, those that measure structural integrity and those that measure functional integrity (National Research Council, 1996). Most indices and methods used by monitoring agencies to evaluate ecological condition have traditionally relied solely on structural indicators such as measurements of biological community composition and habitat structure (Davies and Jackson, 2006; Truchy et al., 2022). Inclusion of functional indicators in condition assessments should enable clearer communications of the status and restoration potential of aquatic ecosystems (Davies and Jackson, 2006) and perhaps better respond to CWA goals. In some cases, functional indicators show a stronger response to stressors than structural indicators and may be able to detect smaller perturbations and thus be more sensitive indicators of ecological integrity (Gulis et al., 2006). A major challenge is identifying and selecting useful indicators because they should be easily measured, be sensitive to the stresses placed on the system under study, respond to the stresses in a predictable manner, have a known response to natural and anthropogenic disturbances, and have low variability in response (Dale and Beyeler, 2001). Ideally, the relationship between indicator and stressor should be linear so that ecological integrity is simply proportional to measured indicator value, although this is often not the case (Young and Collier, 2009). For example, total insect production did not respond in a predictable manner to conductivity along a 5.5-km river continuum; although total insect production was 3.5 times lower in a site with intermediate conductivity (359 μS/cm) compared to an unmined stream (111 μS/cm), total insect production at a higher conductivity site (915 μS/cm) was not different than that of the unmined stream (Voss and Bernhardt, 2017). Further, Vander Vorste et al. (2019) observed that leaf litter decomposition rates were not reduced along a gradient of mining impacted streams with conductivity ranging from 25 to 1,383 μS/cm whereas leaf litter decomposition rates were reduced in MTR-impacted streams with mean conductivities ranging from 1,279 to 3,000 μS/cm (Maxwell, 2009; Fritz et al., 2010).

Odum (1985) hypothesized that certain functional properties are more resistant to stress induced change than are species composition and other structural properties. This greater resistance to stress exhibited by ecosystem functions is due to reserve capacity and/or the ability of some ecosystems to perform these functions by multiple pathways (Bormann, 1985). This functional redundancy is based on variations in genotypes, populations, and species found in many undisturbed or mildly disturbed ecosystems (Bormann, 1985). Small streams draining forested watersheds are largely detritus-based systems with direct litterfall from adjacent vegetation providing ~40% of the energy inputs (Fisher and Likens, 1973). A detritus base is characteristic of relatively mature ecosystems and is thought to confer functional stability to these systems (Odum, 1969). Because clearcutting of existing forest is part of the MTR process, the detritus base of the original system could be decreased to some extent or even temporarily lost. Geogenic organic matter (GOM), which consists of coal fragments and kerogen and is transferred to surface soils because of MTR mining, is a potentially large additional source of organic matter to MTR-impacted streams (Fox, 2009).

The occurrence of MTR coal mining in Central Appalachian watersheds causes changes in stream hydrology, geomorphology (Jaeger, 2015), and physicochemical water quality (Griffith et al., 2012), resulting in changes in structural properties such as altered biofilm bacterial communities (Bier et al., 2015), and decreased macroinvertebrate (Pond et al., 2008), and fish (Hitt and Chambers, 2014) diversity. Most stream assessments of the impact of MTR coal mining to date have employed structural indicators (e.g., Hartman et al., 2005; Lindberg et al., 2011; Ross et al., 2018) with relatively few studies including functional measures (e.g., Fritz et al., 2010; Johnson et al., 2013; Burke et al., 2014; Voss and Bernhardt, 2017; Krenz et al., 2018; Bier et al., 2020).

In this study, we evaluate the impact of land use (forest or MTR coal mining) on structural and functional parameters in perennial streams in the West Virginia coal mining region during fall, winter, spring, and summer sampling campaigns. The structural parameters evaluated as indicators of MTR coal mining included C and nitrogen (N) parameters in water and organic matter content in sediment. Elevated DIC and nitrate concentrations are often observed in MTR-impacted streams, respectively, from the dissolution of carbonates in sedimentary rock layers (Vengosh et al., 2013) and from explosives (Brooks et al., 2019) associated with the MTR process. Ash-free dry mass (AFDM) and dissolved organic carbon (DOC) parameters reflect watershed C sources which may be elevated by C inputs from sedimentary rock layers disturbed during MTR mining (Acton et al., 2011). The functional parameters considered are measures of sediment microbial process rate and biofilm productivity. Fluorescein diacetate activity (FDA), dehydrogenase activity (DHA), denitrification enzyme activity (DEA), and sediment oxygen demand (SOD) are all widely measured indicators of sediment microbial activity, which have not been widely evaluated in streams impacted by MTR mining (but see Burke et al., 2014). DHA in eastern US stream sediments was inversely correlated with alkalinity, conductivity, pH, and sulfate (Hill et al., 2002), which are frequently elevated with MTR mining (Lindberg et al., 2011; Griffith et al., 2012; Ross et al., 2018). FDA associated with freshwater macrophyte decay was shown to be inhibited by increasing conductivity levels (Roache et al., 2006). Higher nitrate concentrations generally lead to higher denitrification activity if redox conditions are suitable and if adequate labile carbon is available (Ambus, 1993; Udy et al., 2006), suggesting DEA as a potential indicator of MTR mining impact. Biofilm production on artificial substrates has been widely used to assess stream water quality (Fellows et al., 2006; Tien et al., 2009). The main purpose of this effort is to: (1) determine which structural and functional indicators in streams responded to MTR coal mining relative to forested controls; (2) determine whether responsive indicators to MTR coal mining responded in a predictable manner; and (3) evaluate whether functional indicators are less likely to change in response to stress than structural indicators.

## Methods

### Study area and sampling approach

**FIGURE 1:**
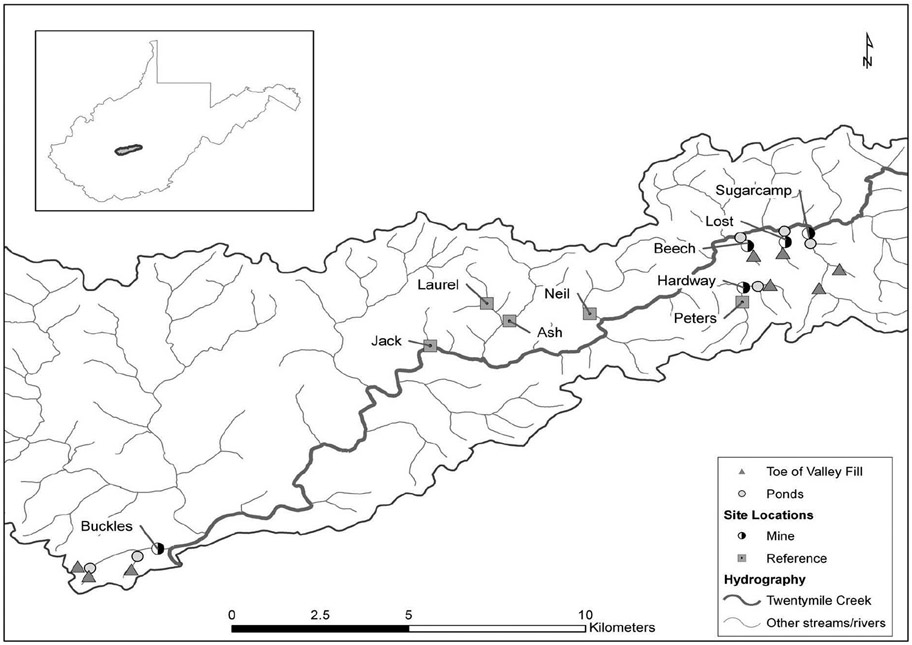
Map of study site locations, sediment ponds, and associated valley fills in the Twentymile Creek watershed, WV, USA (from: Johnson et al., 2013; www.schweizerbart.de/journals/fal).

Ten perennial streams within the Twentymile Creek watershed located in southern West Virginia, USA were chosen for study (Figure 1). The study area consists of steep ridges and narrow valleys and is underlain with middle- and lower-Pennsylvanian cyclic sequences of sandstone, shale, clay, coal, and limestone (Geologic Map of West Virginia; https://www.wvgs.wvnet.edu/www/maps/Geologic_Map_of_West_Virginia-Map25A.pdf). Although carbonates are found deeper in the subsurface, soil surveys found soil pH values generally <6 and no evidence of carbonates in the upper 2 m of forested soils (https://www.nrcs.usda.gov/resources/data-and-reports/web-soil-survey). Further details of the geology, geography, historic land use of the study area, and sampling site locations were described in companion studies conducted at the same sites and time as the present study (Maxwell, 2009; Johnson et al., 2013) and in a previous study (Wiley et al., 2001). Five of the streams drained watersheds (0.57–5.27 km^2^) impacted by MTR mining and five drained areas (0.91–3.99 km^2^) with 95 to 100% mixed hardwood forest cover (Supplementary Table 2). Comparison of 2001 National Land Cover Database (NLCD; https://www.usgs.gov/centers/eros/science/national-land-cover-database) with 2007 aerial photos suggests that most of the mining including creation of valley fills and initial reclamation occurred between 2001 and 2007 (Supplementary Table 3 and Google Earth images in Supplementary material). The MTR-impacted watersheds had 8–62% forest land cover and 38–92% barren land cover, presumably mostly due to MTR coal mining (Supplementary Table 2). All of the forested watersheds were clear-cut in the early 1900s and are impacted by natural gas production, so are not pristine controls, but are representative of the least disturbed condition in the area (Johnson et al., 2013). The streams were evaluated (Johnson et al., 2013) with the Riparian, Channel, and Environmental (RCE) Inventory (Petersen, 1992). The three RCE metrics which describe the riparian zone indicate that, except for one of the mining-impacted streams (Lost), all study streams have reasonably intact riparian vegetation and >80% canopy cover (Supplementary Table 2). All of the forested streams had total RCE scores in the “Excellent” class, four of the MTR-impacted streams scored as “Very good” and one MTR-impacted stream (Lost) scored as “Good” (Johnson et al., 2013). A companion study conducted in these sites at the same time as the present study found substantial sediment accumulation in litterbags deployed in the MTR-impacted streams suggesting considerable mobilization of sediment by the MTR mining process (Maxwell, 2009). Sediment metrics associated with the RCE inventory and the Rapid Bioassessment Protocol (RBP; Barbour et al., 1999) assessments conducted by Johnson et al. (2013) and reported in Supplementary Table 2 also suggest sedimentation impacts in the MTR-impacted streams.

The parameters described here were collected from 3 to 7 times between October of 2007 and July of 2008. Water samples were collected from the main flow path at middepth. Conductivity, temperature, and pH of stream water were measured in a companion study (Johnson et al., 2013) with a portable multiprobe (Hydrolab Quanta; Hydrolab Corp., Austin, TX) and are reported in Supplementary Table 4. Sediment samples were collected from the upper 2 cm of about five depositional areas within the wetted area of the channel along 100 m reaches and composited in each study stream. Sediments that passed through a 2-mm sieve were collected and further processed. For the biofilm production studies, two cinder blocks (20.3 × 40.6 × 4.2 cm), each with 25 fused-silica porous disks (5.31 cm^2^, Leco Corporation #528-042) attached were deployed on the stream beds in conjunction with the seasonal sampling trips and allowed to colonize for 9–36 days.

### Structural measures

All water samples for DOC and DIC analyses were filtered in the field with GF/F syringe filters (0.7 μM pore size) and collected in 40-ml amber volatile organic analysis vials. Sample vials for DOC were capped with Teflon septa, and those for DIC were carefully filled to overflowing to exclude air and capped with butyl rubber septa. Water samples were transported to the lab on ice and refrigerated until analysis. The concentration and stable carbon isotopic composition (δ^13^C) of DOC and DIC were determined as previously described (Burke et al., 2014). Briefly, an OI Analytical 1030 W total inorganic carbon-total organic carbon analyzer (OI Analytical, College Station, TX) interfaced to a Thermo Delta V Plus isotope ratio mass spectrometer (Thermo Fisher Scientific, Waltham, MA) was used for DOC and DIC analyses. DOC parameters were analyzed following acidification and persulfate digestion and DIC parameters were analyzed following acidification. Stable carbon isotope ratios are expressed as per mil (‰) in the delta notation vs. Vienna Peedee Belemnite (VPDB):

$$\delta^{13}C=[[{(}^{13}C{∕}^{12}C)\;sample∕{(}^{13}C{∕}^{12}C)\;standard]-1]\;x\;1000$$

All reported DIC and DOC concentration and δ^13^C values are based on at least two measurements. Dissolved CO_2_ concentrations, expressed as CO_2_ partial pressure (pCO_2_) in μatm, were calculated using the CO_2_Calc package (Robbins et al., 2010) from DIC concentrations and corresponding pH and temperature measured in the field. Nitrate concentrations in field-filtered (GF/F) water samples were measured by second-derivative UV spectroscopy (Crumpton et al., 1992). The AFDM of two sediment size fractions associated with different functional measures were determined by combustion at 500°C for 5 h. The AFDM of sediments that passed a 2-mm sieve will be referred to as AFDM2. A swirl-decantation technique (Palmer, 1990) was used in the field to obtain a fine sediment fraction from the 2 mm-sieved sediments. Briefly, 2 mm-sieved sediments were placed in a tub with stream water and the mixture was swirled around to suspend a finer and/or less dense fraction, which was then poured off into a cup. This suspended sediment fraction will be referred to here as “fine sediment” and AFDM of this fine sediment fraction will be referred to as AFDMf. AFDM was determined on all sediment sample replicates analyzed for a functional indicator except for DHA. For DHA, AFDMf was based on analyses of a single 10-g composite sediment sample collected from each stream on each date as described above. Although AFDM2 and AFDMf were each analyzed in two separate sets of samples, they are considered to be two indicators rather than four. All samples for AFDM analysis were put on ice, frozen as soon as possible, and transported to the lab.

### Functional measures

#### Sediments

Denitrification is the microbial respiration of nitrate to nitrogen gas (N_2_) or nitrous oxide (N_2_O), using nitrate as the electron acceptor (Payne, 1973). Sediment DEA was measured in the lab by incubating ~25 g of 2 mm-sieved sediment, and 25 ml of a solution consisting of 100 mg N/L of nitrate, 40 mg/L of dextrose, and 10 mg/L of chloramphenicol dissolved in high purity water in an atmosphere of ~10% acetylene/90% nitrogen, for ~1.5h, with gas samples generally taken at 30 min and 1.5 h (Groffman et al., 1999). The DEA assay provides conditions that are non-limiting for denitrification (anaerobic, abundant nitrate and labile carbon), but which inhibit organism growth (chloramphenicol). DEA is thus an estimate of the maximum potential denitrification rate by the denitrifying community present at the time of sampling.

Sediment denitrification rate (DeN) was measured similarly to DEA but using site water with 10 mg/L of chloramphenicol and no added nitrate or labile carbon and with an ~2 h incubation time, and gas samples generally taken at 30 min and 2 h (Loken et al., 2016). DEA and DeN were estimated by measuring production of N_2_O, which is the final product of denitrification with the added acetylene, during incubation. Acetylene is known to inhibit nitrification, so coupled nitrification/denitrification, which is the major source of N for denitrification when the overlying water has a nitrate concentration <140 μg N/L (Seitzinger et al., 2006) is also inhibited. As a result, DeN is likely lower than the actual *in situ* denitrification rate when stream nitrate concentrations are low. At overlying water nitrate concentrations in excess of 840 μg N/L, nitrate in the overlying water accounts for about 80% of the N used in denitrification (Seitzinger et al., 2006) and DeN could approximate actual *in situ* denitrification rates. All reported DEA and DeN values are based on measurement of at least two separate subsamples.

Sediment oxygen demand (SOD) includes the aerobic respiration of all organisms living in the sediment and chemical oxidation of reduced species such as Fe (II), Mn (II), and sulfide (Bowman and Delfino, 1980). SOD was estimated in the field as the change in oxygen concentration, as measured with a YSI Model 58 m and Model 5905 stirring dissolved oxygen (DO) probe (YSI Inc., Yellow Springs, OH) in 50-ml centrifuge tubes after incubation. The centrifuge tubes were completely filled with ~5 g of 2 mm-sieved sediment and stream water and incubated in the dark for ~2 h at ambient stream temperature (Hill et al., 2002). The SOD data are calculated based on measurement of five replicates and two stream water blanks.

Fluorescein diacetate is hydrolyzed to fluorescein by a wide range of intracellular and extracellular enzymes involved in organic matter decomposition, including esterases, proteases, and lipases, and it is generally assumed that the FDA assay provides a synoptic estimate of microbial decomposer activity (Adam and Duncan, 2001). Sediment FDA was measured (Green et al., 2006) by mixing ~0.5 g of fine sediment with 5 ml of phosphate buffer (pH 7.6) and 0.5 ml of fluorescein diacetate solution (8.4 mM) in 50-ml centrifuge tubes in the field, incubating for 30 min in the dark at ambient stream temperature, and terminating the hydrolysis reaction with acetone. The reacted samples were put on ice, frozen as soon as possible, and transported to the lab. After thawing and centrifugation, the fluorescein concentration of the supernatant was determined at 490 nm by spectrophotometry. The sediment FDA data are calculated based on triplicate measurements with a single reagent control.

Dehydrogenase activity (DHA), which is often referred to as electron transport system (ETS) activity in the literature (e.g., Trevors, 1984; Blenkinsopp and Lock, 1990), estimates the activity of intracellular hydrogenases that catalyze the redox reactions required for organic matter decomposition and is correlated with microbial activity (García et al., 1994). Sediment DHA was measured in the lab by adding ~0.5 g of fine sediment, 4 ml of deionized water, and 1 ml of 0.14% iodonitrotetrazolium chloride (INT) solution to a 50-ml centrifuge tube and vortexing for 30 s. The samples were then incubated for 6 h at 36°C, centrifuged for 5 min at 3,000 rpm, the supernatant removed, and 8 ml methanol added. The samples were again vortexed for 30 s, the supernatant removed, and the INT-formazan (INTF) produced was quantified at 440 nm by spectrophotometry. The sediment DHA data are triplicate measurements with duplicate controls that consisted of sediment and high purity water without any INT addition. The data presented here can be thought of as an estimate of *in situ* DHA because ETS stimulators (NADH, NADPH, and succinate) were not added so that disruption of sediment biofilms could be avoided (Blenkinsopp and Lock, 1990). The DHA values presented here are thus lower than many measurements of potential DHA from the literature that were determined using methods (e.g., Broberg, 1985) that added ETS stimulators. Sediment functional measurements are expressed on a g per dry mass (DM) or g AFDM basis (either AFDM2 or AFDMf).

#### Biofilm colonization

One or two disks, collected from the stream each visit, were incubated in 50-ml centrifuge tubes to measure biofilm production of oxygen demand, FDA, DHA, and chlorophyll *a*. Biofilm oxygen demand was measured with the same technique that was used for SOD. Biofilm FDA and DHA analyses used the same reagent additions, reaction times and temperatures, and spectrophotometry techniques as were used for the sediment analyses. Chlorophyll *a* was analyzed (Lorenzen, 1967; Sartory and Grobbelaar, 1984) by adding 10 ml of 90% ethanol to each tube and ensuring that the disks were completely submerged, shaking, incubating for 5 min at 78°C, and allowing tubes to stand in the dark at room temperature for 24–72 h. The extracts were then poured into other tubes, centrifuged for 20 min at 2,000 rpm, the supernatants removed and measured at 750, 664, 647, and 630 nm without acid and then at 750 and 665 nm after addition of 0.155ml of 0.2N HCl and a 90s reaction period. Chlorophyll *a* amount was calculated as previously described (Lorenzen, 1967) with modifications. Production rates of biofilm oxygen demand (BfOD), biofilm FDA (BfFDA), and biofilm DHA (BfDHA) were calculated by dividing incubation rates by disk area and the colonization period length (days). Biofilm chlorophyll *a* production rate (BfChla) was calculated by dividing chlorophyll *a* mass by disk area and the colonization period length. BfOD, BfFDA, and BfDHA data were based on the same number of replicates and blanks as for the corresponding sediment parameters. BfChla data were based on triplicate measurements with blanks scattered throughout each sample set.

### Data analysis

The Number Cruncher Statistical Software (NCSS) NCSS2019 package (NCSS, Kaysville, UT) was used for statistical analyses. The NCSS2019 Mixed Models procedure was used for repeated measures comparisons of each structural and functional parameter measured to determine if these parameters responded to MTR coal mining relative to forested controls. Sampling date was the time variable and the Kenward-Roger adjustment for degrees of freedom was applied to all analyses. Models with a range of error covariance structures (R matrices) of varying complexities were run and the model with the lowest Akaike Information Criteria (AIC) value was accepted (Anderson et al., 2000). Because the number of observations never exceeded the number of model parameters by at least a factor of 40, a modified criterion for small ample size correction called AICc was used for model selection (Anderson et al., 2000). To determine whether responsive indicators to MTR coal mining responded in a predictable manner multiple comparisons tests [Least Squares Means (LS Means) with Bonferroni adjustments] were run to identify specific differences when significant differences among treatments were indicated. Mean values of the dependent variables across replicates collected from a reach during a specific sampling trip were treated as the statistical unit for comparisons. Means reported in the text, tables, and figures are LS Means calculated by the NCSS2019 software. Results of the mixed models with the lowest AICc for each of the parameters reported here are given in Tables 1, 2, and Supplementary Table 5. The NCSS Linear Regression and Correlation routine was used to test for linear relationships between the functional indicators and temperature, conductivity, and pH when data from all dates and sampling sites were combined.

We used Miller-Tans plots of our DIC concentration and stable carbon isotopic composition (δ^13^C-DIC) data to estimate the source δ^13^C-DIC to forested and mining-impacted streams (Miller and Tans, 2003). The Miller-Tans plot (δ^13^C-DIC * DIC concentration vs. DIC concentration) results from an equation derived such that (1) the slope provides an estimate of the δ^13^C of a source (e.g., CO_2_ from soil respiration or DIC from carbonate dissolution) mixing with a background component (e.g., atmospheric CO_2_); and (2) the concentration and δ^13^C of the background component need not be known and can be variable (Miller and Tans, 2003). The slopes of the Miller-Tans plots were determined with the linear regression procedure in the NCSS2019 software.

We assessed the possible influence of CO_2_ degassing on DIC concentration and δ^13^C-DIC by performing degassing simulations (Venkiteswaran et al., 2014). Degassing is relatively more important in low-productivity streams draining silicate-dominated watersheds with low to moderate pH values (<7) and high CO_2_ concentrations, such as the forested streams studied here (Johnson et al., 2013; Venkiteswaran et al., 2014). For the degassing simulations we assumed a soil CO_2_ concentration of 20,000 μatm and a source δ^13^C-DIC of −22.3‰ (value calculated with the Miller-Tans plot of the forested stream data—see below) for the soil respiration source. We simulated degassing by removing portions of the CO_2_, initially in increments of 1,000 μatm down to 10,000 μatm, then in increments of 500 μatm down to 2,500 μatm, and finally in increments of 100 μatm down to 300 μatm. The CO_2_Calc package was used to calculate the redistribution of carbonate species and the pH change with degassing with constant carbonate alkalinity (Robbins et al., 2010). We imported the information regarding carbonate species changes with degassing into an Excel spreadsheet, which was used to perform all further calculations. We applied carbon isotopic fractionations of 2 and 3.5% (Wanninkhof, 1985; Zhang et al., 1995), respectively, to the increments of degassed CO_2_ and used the approach described by Campeau et al. (2017) and the data from CO2calc to calculate δ^13^C-DIC changes resulting from degassing.

## Results

### Structural measures

**FIGURE 2:**
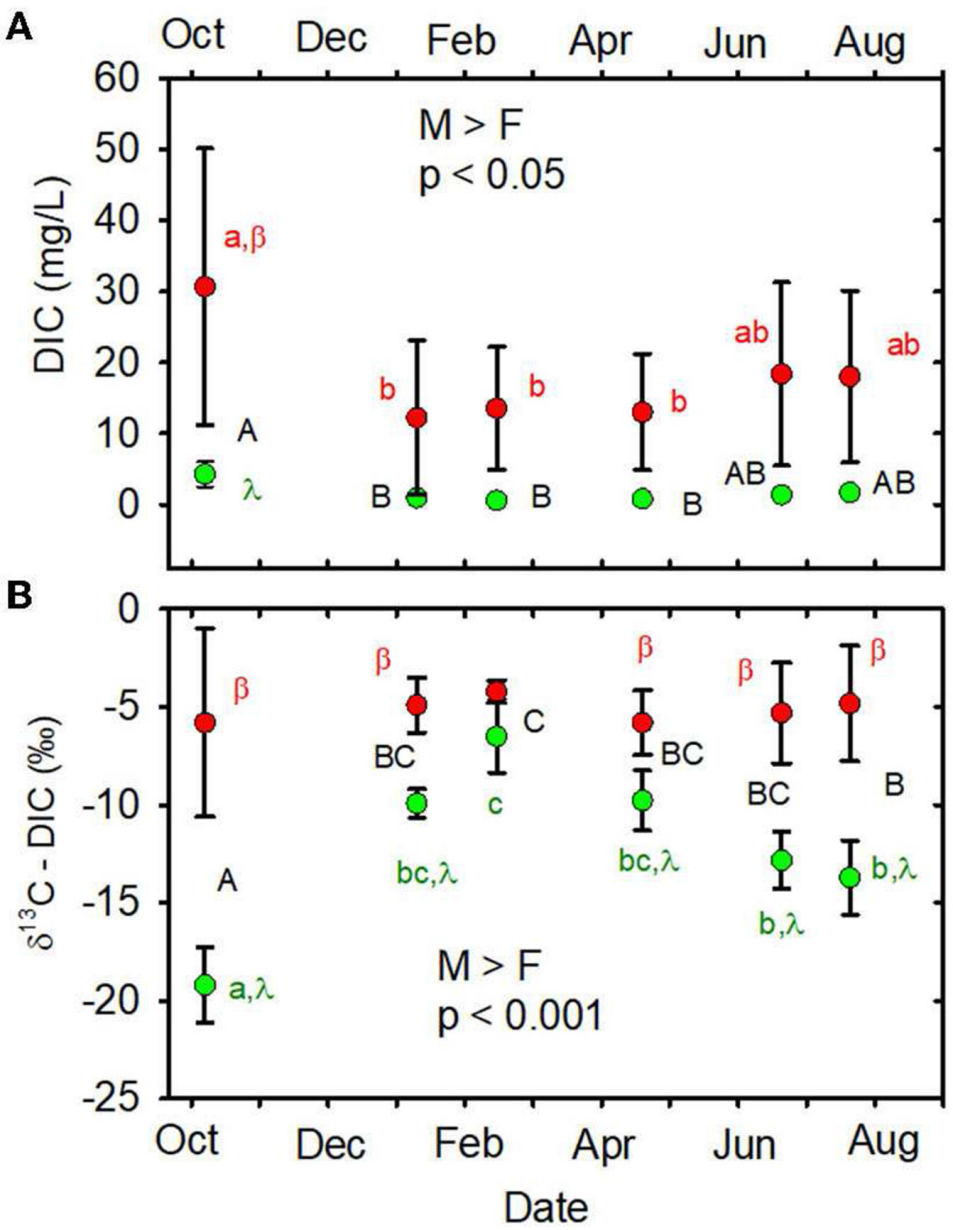
**(A)** DIC concentration (mg/L) vs. date. **(B)** δ^B^C-DIC (‰) vs. date. Statistical differences across all dates and sites are indicated as M > F or F > M and *p*-value. Different capital letters indicate significant time effects across all sites, different lowercase letters indicate significant time effects within a given land use, and Greek letters indicate a significant land use impact at a given time. When letters are not shown, no significant differences were observed. Forested watersheds — 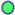, MTR watersheds — 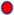.

When data from all sampling dates were combined, mean DIC concentration was greater in the MTR-impacted streams than in the forested streams (Table 1, Figure 2A). Time and land use * time interactions are indicated on Figure 2A and in Supplementary Table 5. DIC concentrations varied a lot less in the forested streams than in the MTR-impacted streams (Figure 2A, Supplementary Table 6).

**FIGURE 3:**
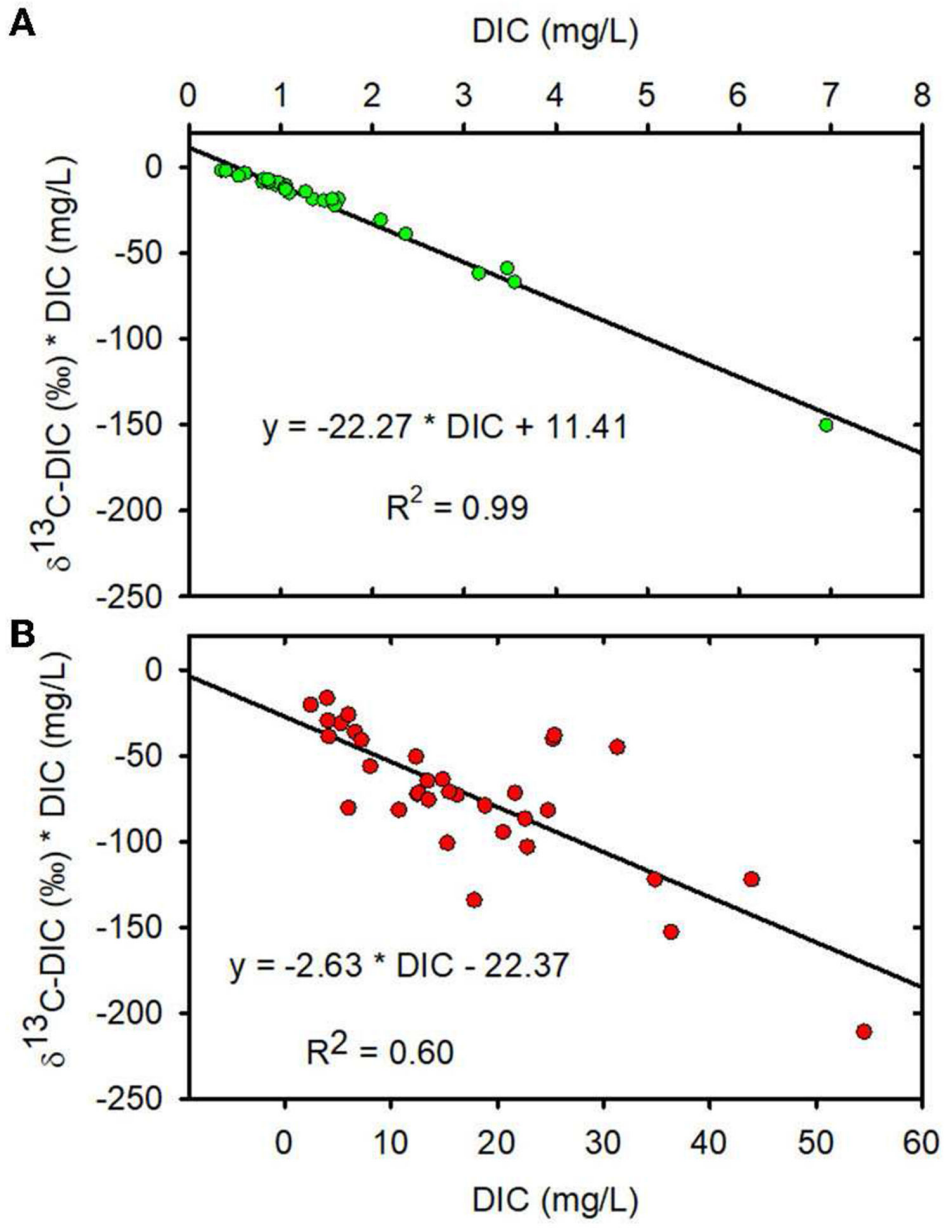
**(A)** Miller-Tans plot for forested watersheds. **(B)** Miller-Tans plots for mined watersheds. Forested watersheds — 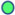, MTR watersheds — 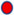.

When data from all sampling dates were combined, mean DIC stable carbon isotopic composition (δ^13^C-DIC) was greater in the MTR-impacted streams than in the forested streams (Table 1, Figure 2B). The Miller-Tans plots are presented in Figures 3A, B and suggest watershed-source inorganic carbon δ^13^C values of −22.3 and −2.6‰, respectively, for the forested and MTR-impacted streams.

**FIGURE 4:**
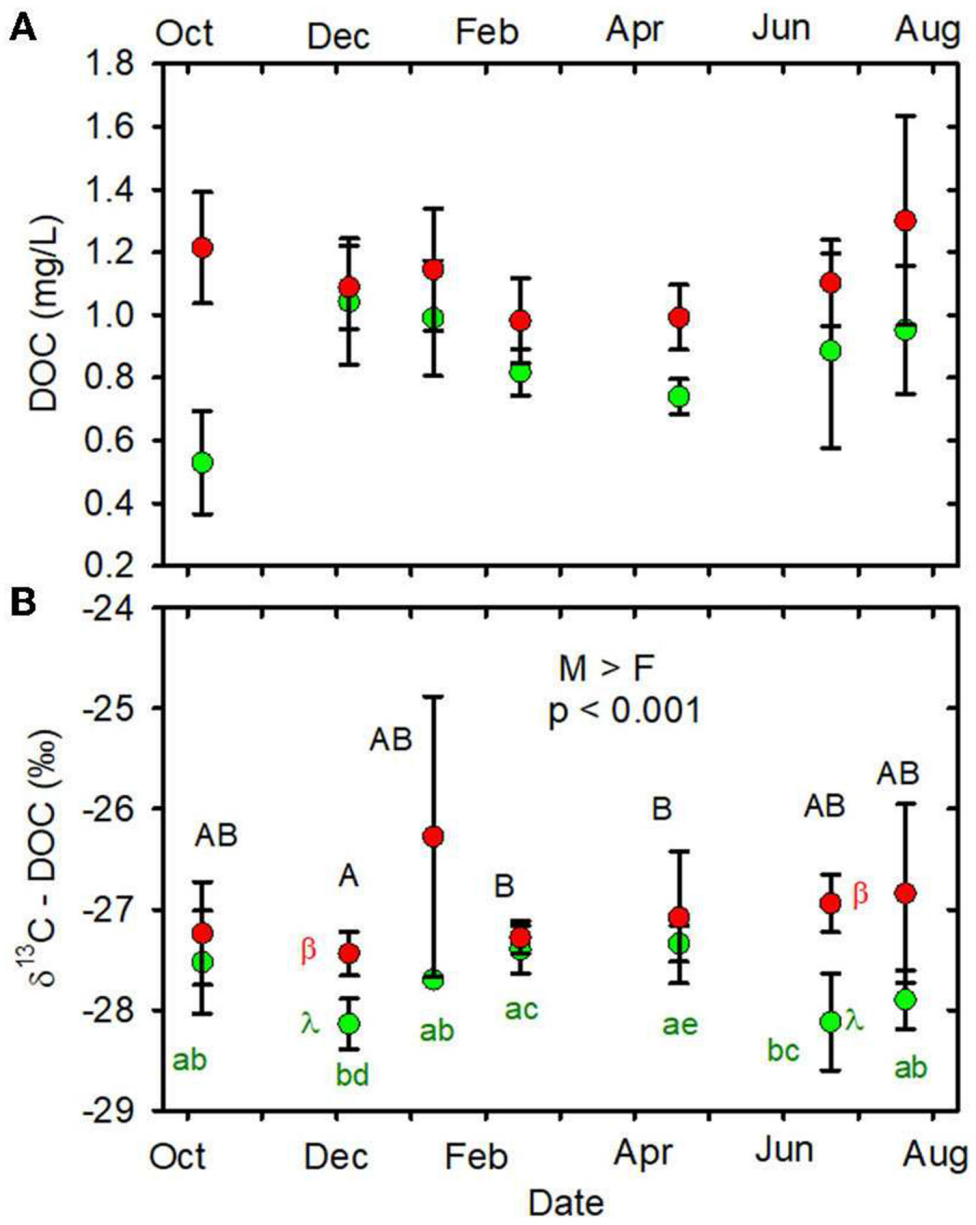
**(A)** DOC (mg/L) vs. date. **(B)**: δ^13^C-DOC (%) vs date. Statistical differences indicated as in Figure 2. Forested watersheds — 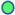, MTR watersheds — 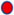.

When data from all sampling dates were combined, the mean DOC stable carbon isotopic composition (δ^13^C-DOC) in the MTR-impacted streams was greater than the mean in the forested streams (Table 1, Figure 4B, Supplementary Table 6).

**FIGURE 5:**
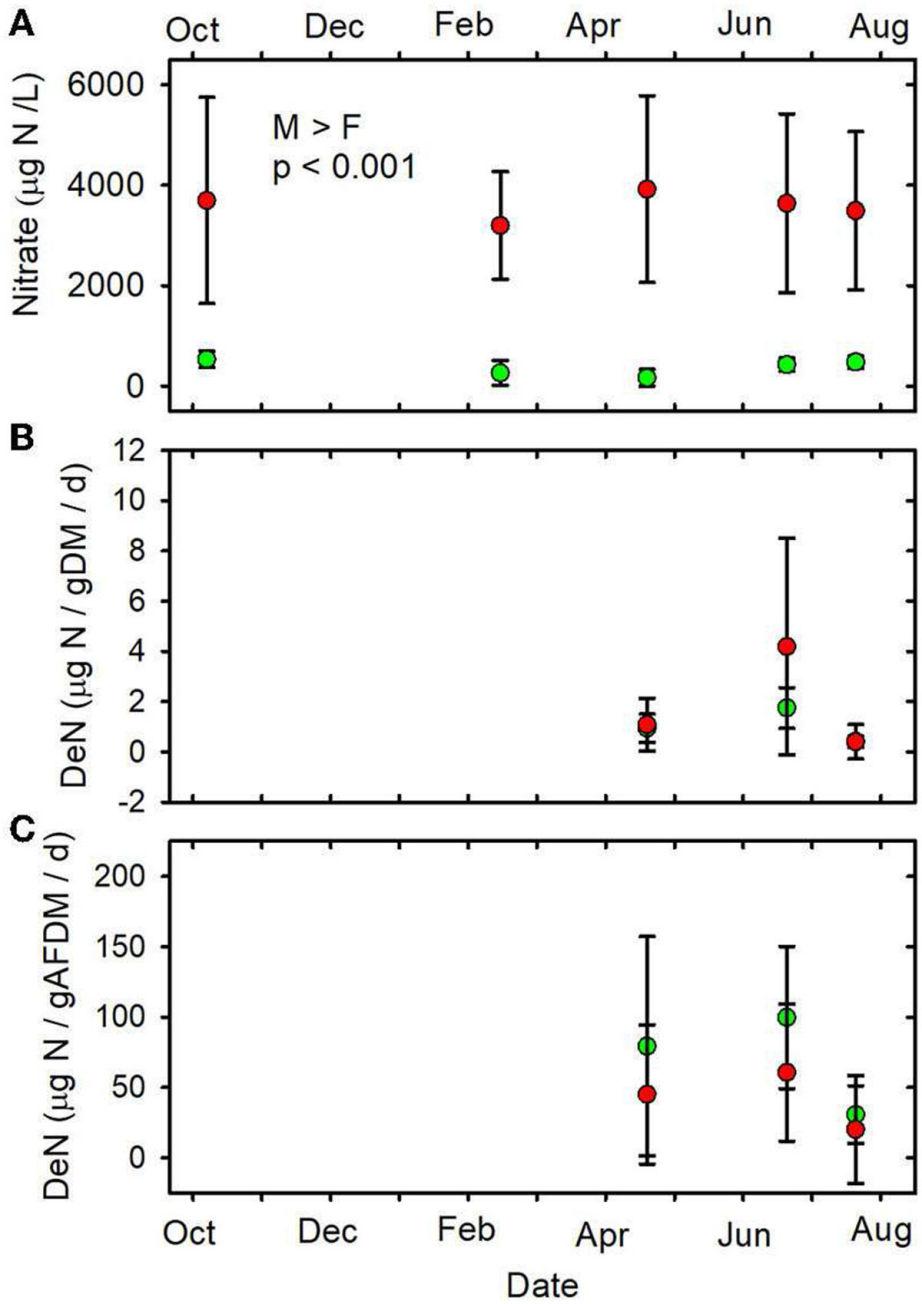
**(A)** Nitrate concentrations vs. date. **(B)** DeN/gDM vs. date. **(C)** DeN/gAFDM vs. date. Statistical differences indicated as in Figure 2. Forested watersheds — 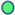, MTR watersheds — 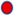.

The mean nitrate concentration in the MTR-impacted streams was almost 10 times higher than in the forested streams (Table 1, Figure 5A, Supplementary Table 7). Mean nitrate concentrations were higher in the MTR-impacted streams than in the forested streams on every date and there were no significant seasonal variations (Table 1, Figure 5A). Nitrate concentrations were much less variable in the forested streams than in the MTR-impacted streams (Figure 5A).

AFDMf values were considerably higher than AFDM2 values suggesting that the swirl-decantation technique (FDA and DHA) preferentially removed an organic-rich sediment fraction from the 2 mm-sieved sediment (Table 1, Supplementary Table 8). Mean AFDM2-DEA was very similar to mean AFDM2-SOD in both forested and MTR-impacted streams (Table 1). Both AFDM2-DEA and AFDM2-SOD means were greater in the MTR-impacted streams than in the forested streams when data from all sampling dates were combined (Table 1).

**FIGURE 6:**
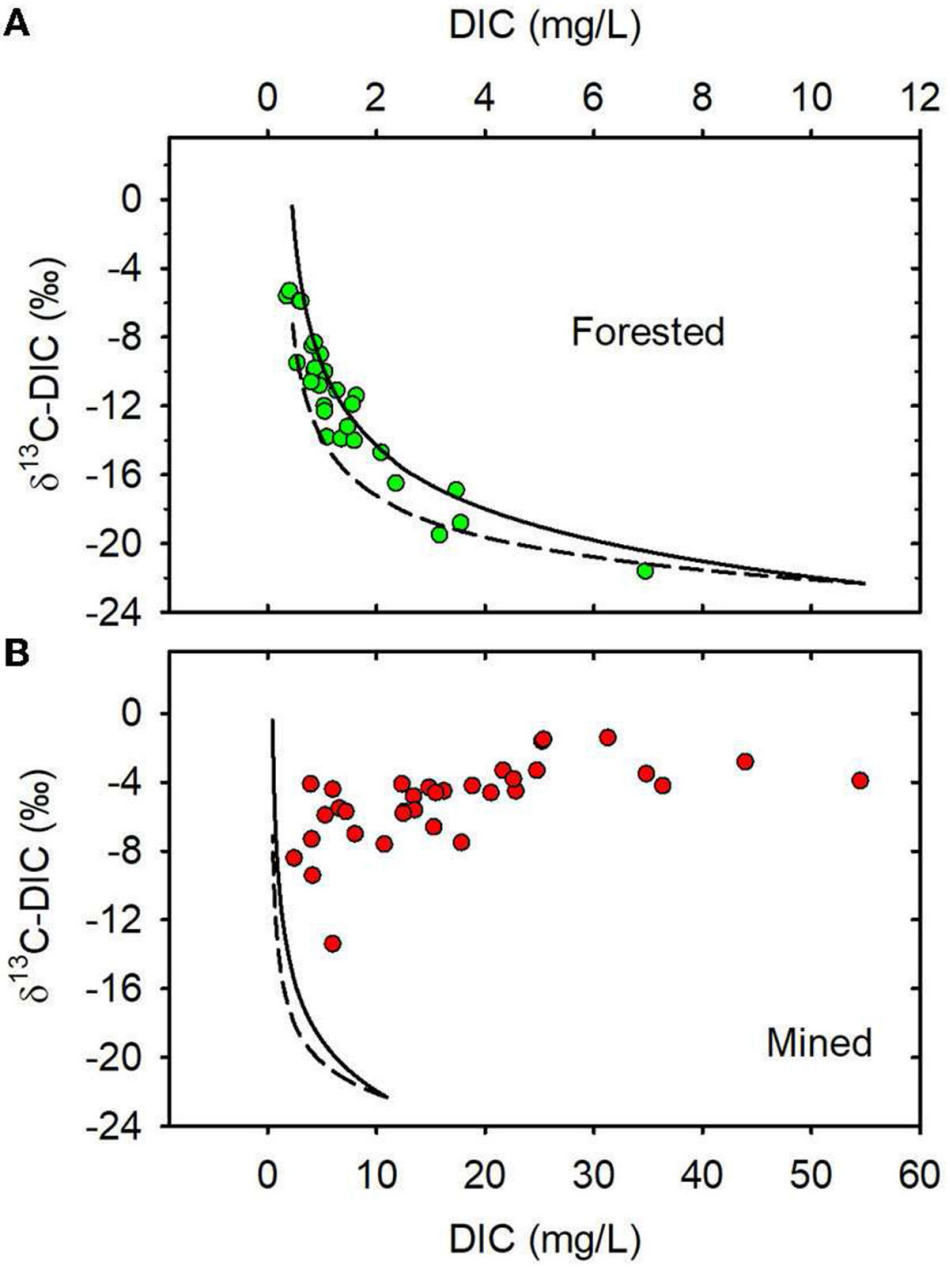
**(A)** Degassing simulation for forested watersheds. **(B)** Degassing simulation for mined watersheds. Solid line is 3.5% isotopic fractionation and dashed line is for 2% isotopic fractionation. Forested watersheds — 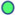, MTR watersheds — 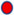.

When data from all sampling dates were combined, mean DOC concentration (Figure 4A), mean pCO_2_ (Supplementary Table 7), and mean AFDMf (Supplementary Table 8) were not different between MTR-impacted and forested streams (Table 1). The pCO_2_ of all but two of our samples (Supplementary Table 7) exceed the mean 2008 atmospheric pCO_2_ of 385 μatm (Lan et al., 2022) indicating that these streams are generally expected to degas CO_2_ to the atmosphere. The pH values of the two samples from Sugarcamp with sub-atmospheric levels of CO_2_ were high enough (8.8 and 8.9; Supplementary Table 4) to shift the carbonate equilibria away from CO_2_ toward ${\text{HCO}}_{3}^{-}$ and ${\text{CO}}_{3}^{=}$ (Zeebe et al., 1999). The results of the degassing simulation are presented in Figures 6A, B.

Five of the eight structural parameters measured were detectably different between MTR-impacted and forested streams. Means for these five structural parameters were greater in the MTR-impacted streams than in the forested streams across all sampling dates. This difference (MTR > forested) was more consistently observed on individual sampling dates for δ^13^C-DIC (5 dates) and nitrate (5 dates) than for δ^13^C-DOC (2 dates), DIC concentration (1 date), or AFDM2 (no dates; Supplementary Table 5).

### Functional measures

**FIGURE 7:**
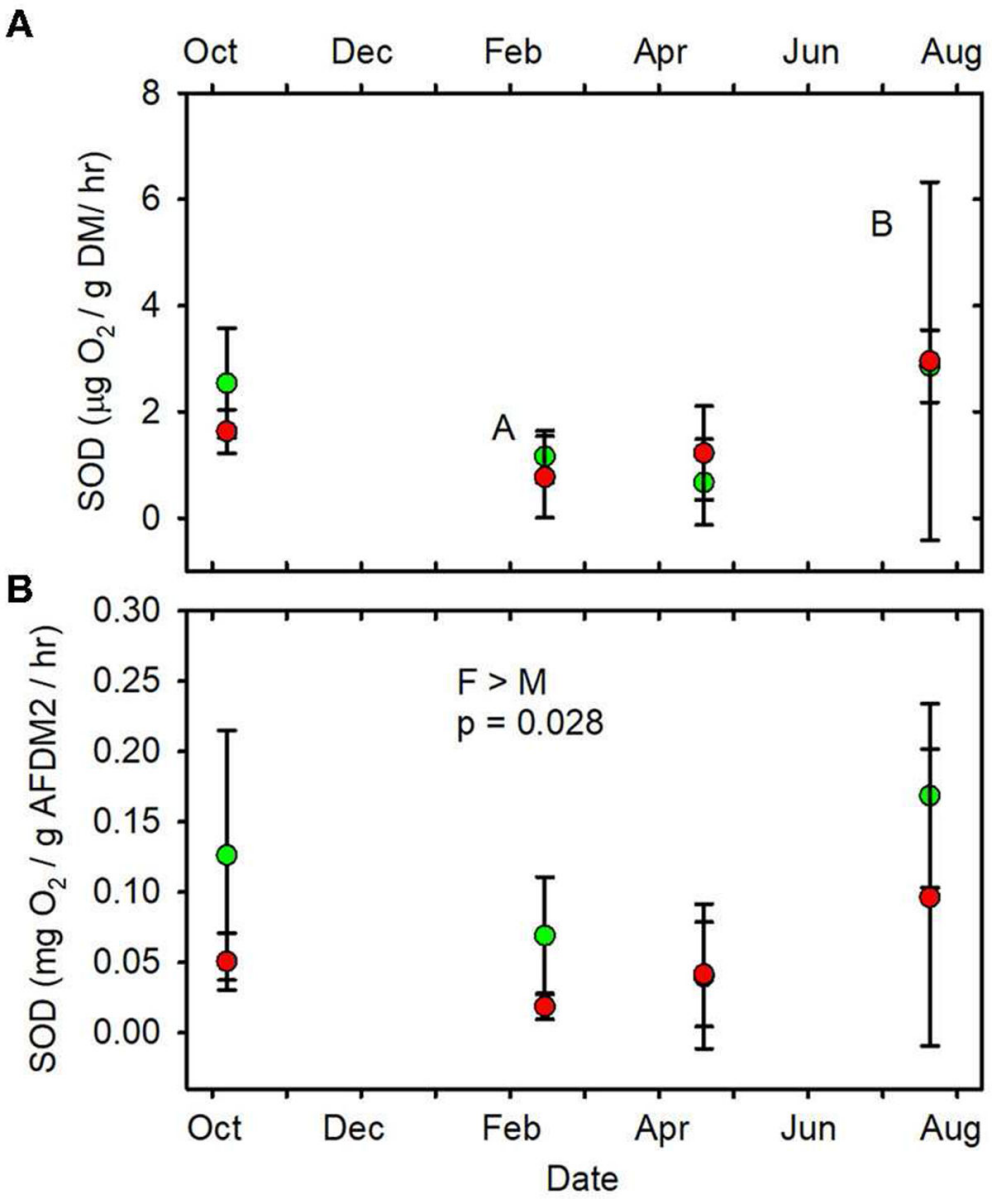
**(A)** SOD/gDM/hr vs. date. **(B)** SOD/gAFDM2/hr vs. date. Statistical differences indicated as in Figure 2. Forested watersheds — 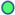, MTR watersheds — 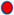.

**FIGURE 8:**
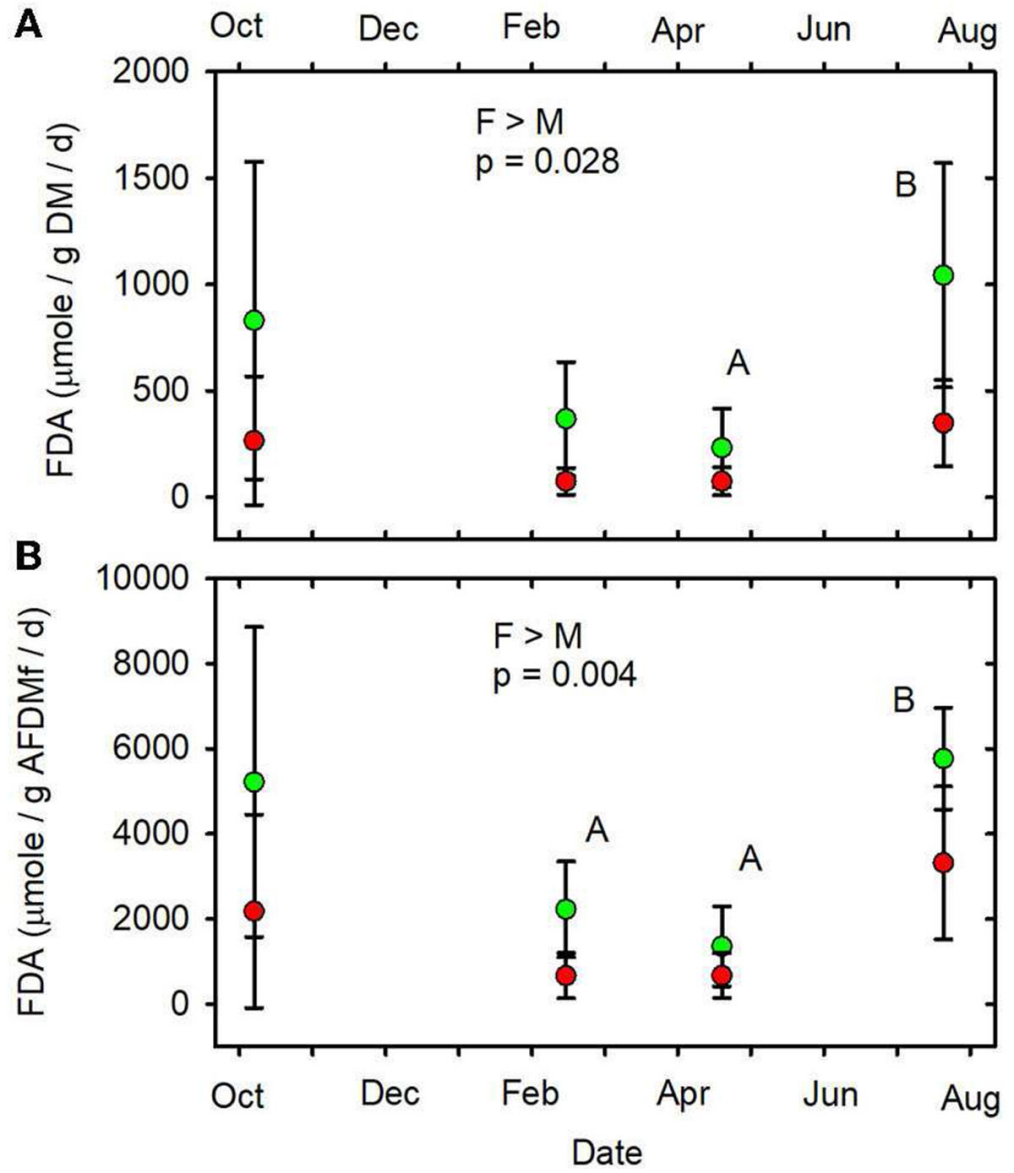
**(A)** FDA/gDM/d vs. date. **(B)** FDA/gAFDMf/d vs. date. Statistical differences indicated as in Figure 2. Forested watersheds — 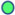, MTR watersheds — 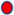.

When data from all sampling dates were combined, mean SOD/gAFDM2 (Figure 7B, Supplementary Table 9), mean FDA/gDM (Figure 8A, Supplementary Table 10) and mean FDA/gAFDMf (Figure 8B, Supplementary Table 10) were greater in the forested streams than in the MTR-impacted streams (Table 2).

**FIGURE 9:**
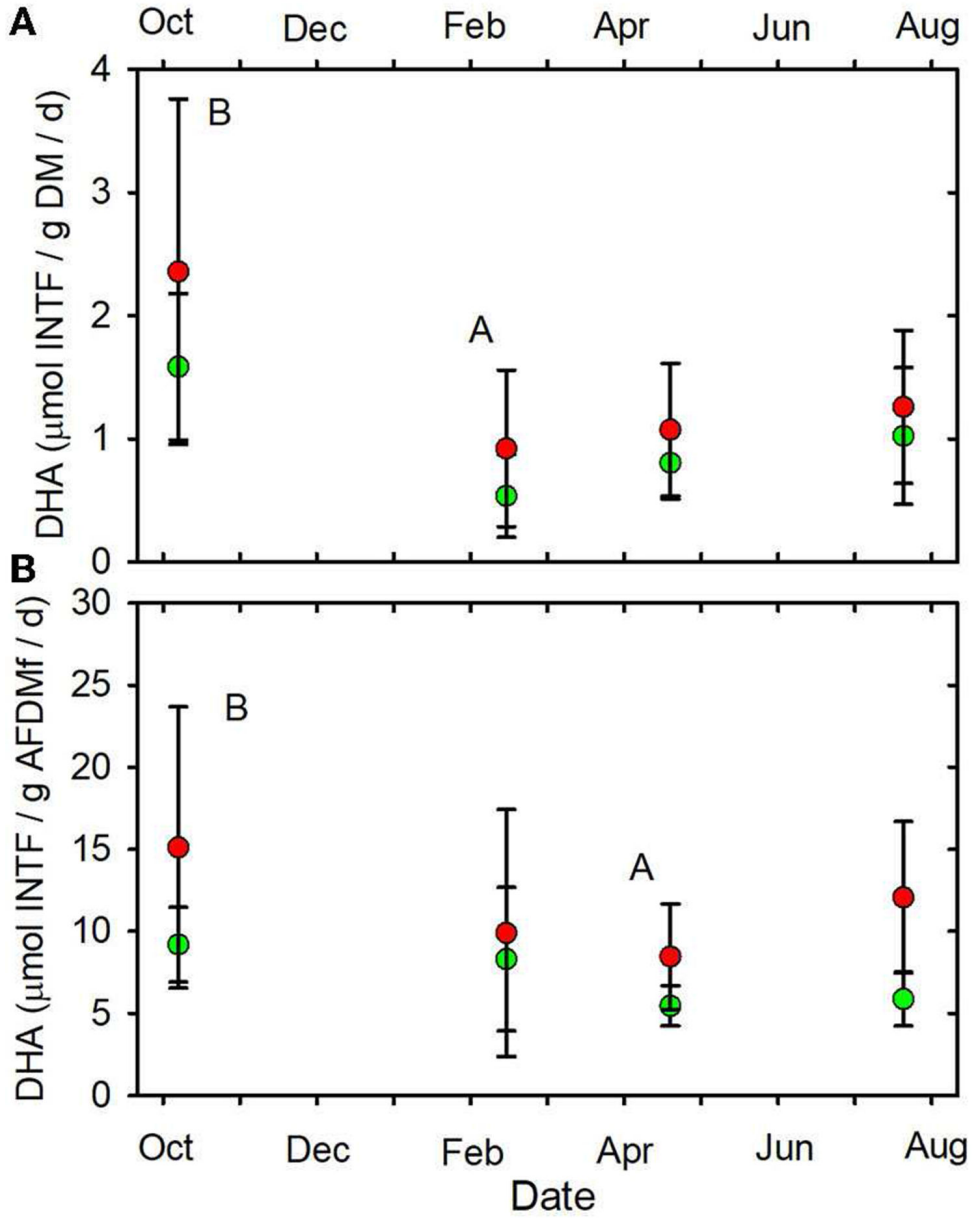
**(A)** DHA/gDM/d vs. date. **(B)** DHA/gAFDMf/d vs. date. Letters indicating statistical differences indicated as in Figure 2. Forested watersheds — 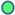, MTR watersheds — 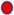.

**FIGURE 10:**
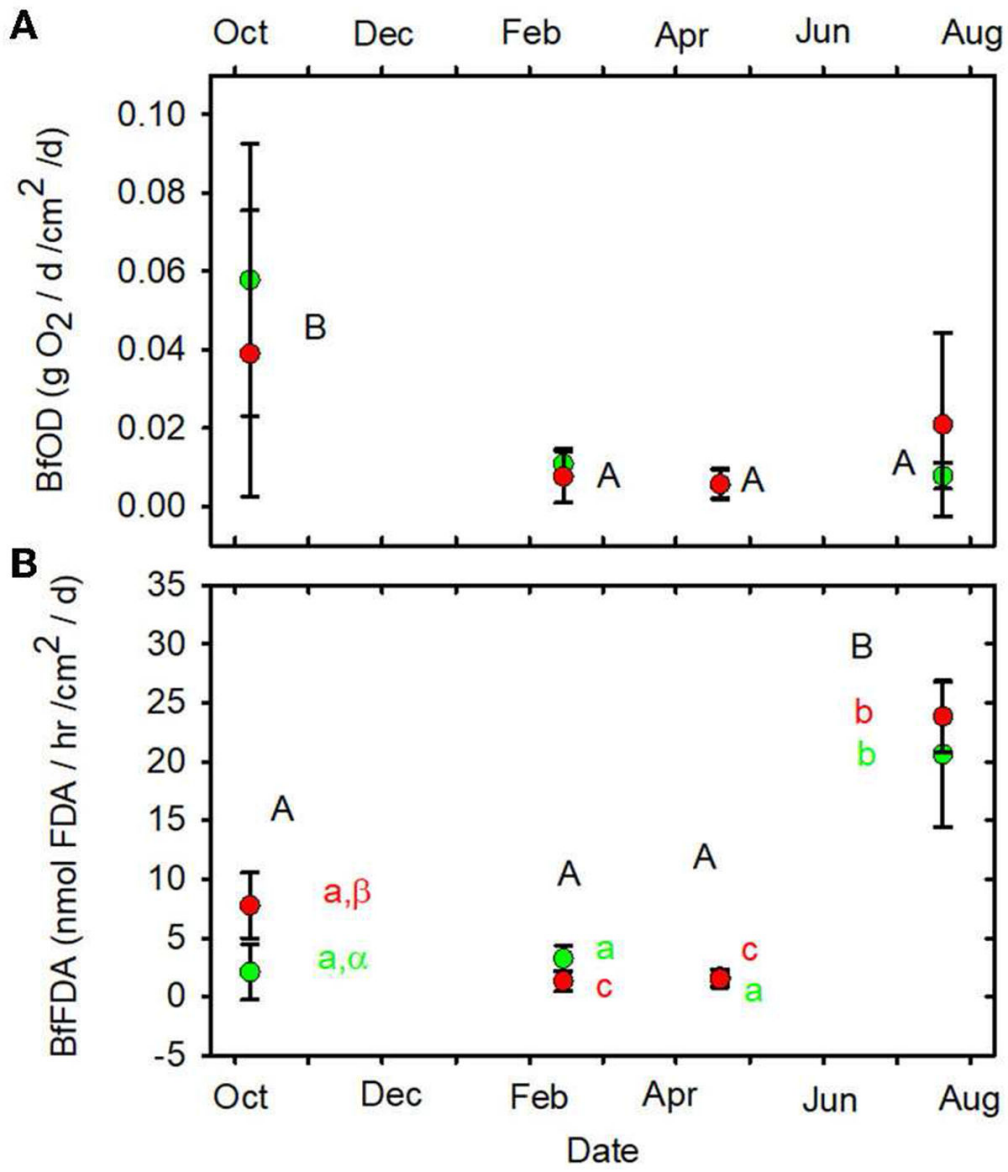
**(A)** BfOD vs. date. **(B)** BfFDA vs. date. Statistical differences indicated as in Figure 2. Forested watersheds — 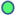, MTR watersheds — 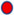.

**FIGURE 11:**
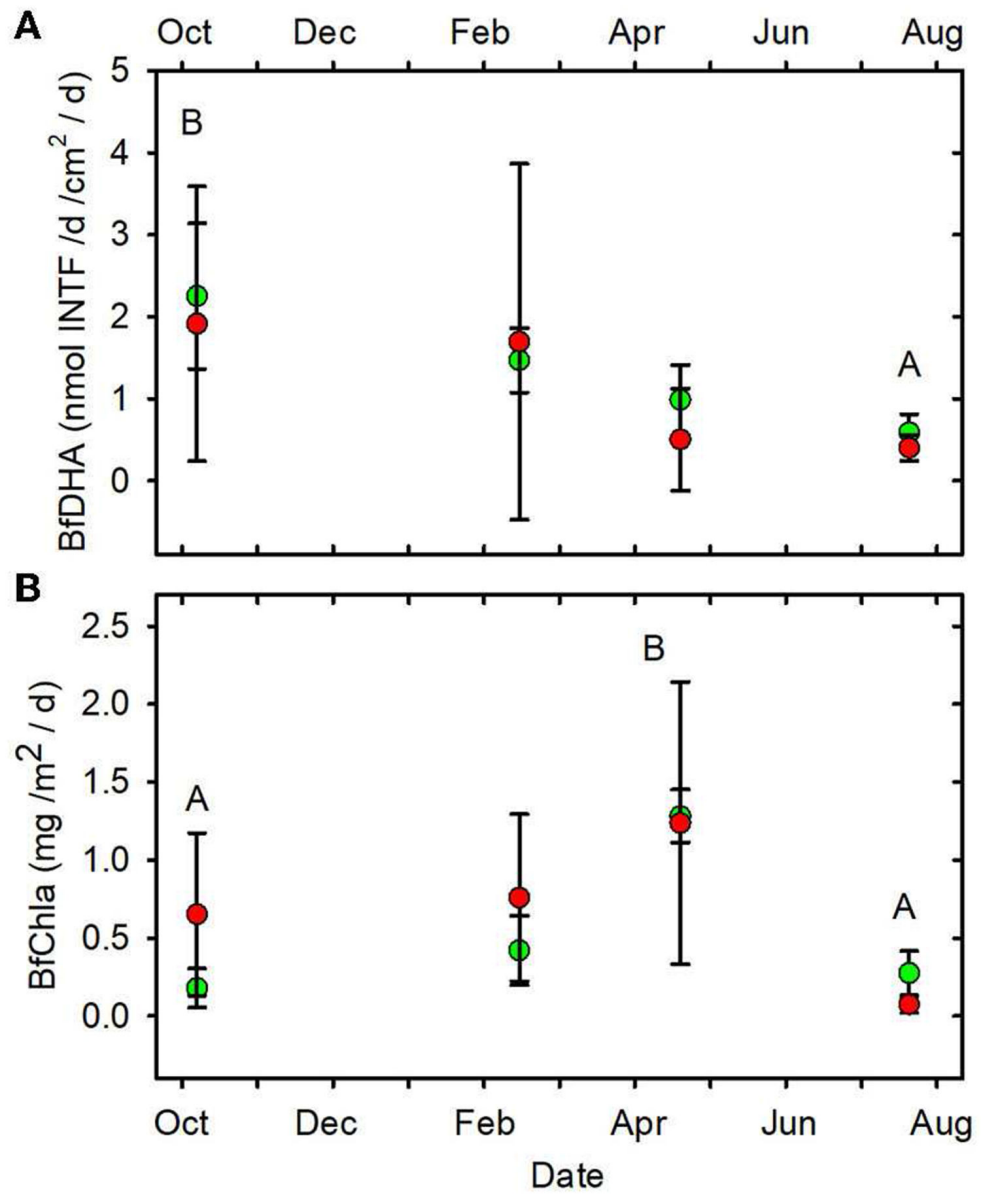
**(A)** BfDHA vs. date. **(B)** BfChla vs. date. Statistical differences indicated as in Figure 2. Forested watersheds — 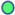, MTR watersheds — 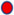.

**FIGURE 12:**
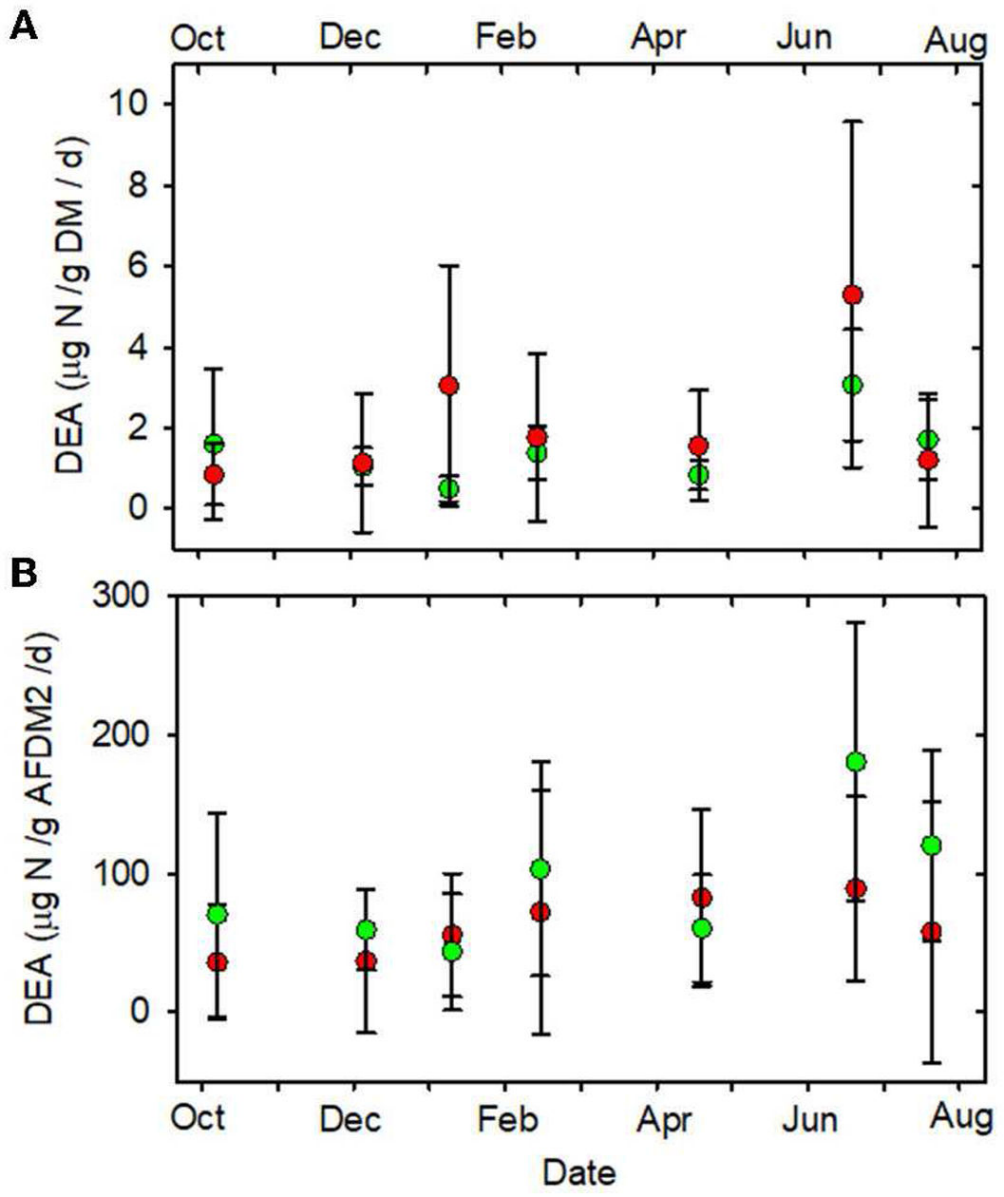
**(A)** DEA/gDM/d vs. date. **(B)** DEA/gAFDM2/d vs. date. Statistical differences the same as in Figure 2. Forested watersheds — 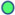, MTR watersheds — 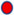.

None of the other functional indictors differed between the forested and MTR-impacted streams across all sampling dates, although there were significant time differences and BfFDA exhibited a significant land use * time interaction (Supplementary Table 5). When data from all sampling sites were combined, summer-fall (June, July, October) mean values of SOD/gDM (Figure 7A), FDA/gDM (Figure 8A), FDA/gAFDM (Figure 8B), DHA/gDM (Figure 9A), DHA/gAFDM (Figure 9B), BfOD (Figure 10A) BfFDA (Figure 10B), and BfDHA (Figure 11A) were higher than values in winter-spring (December-April; Supplementary Table 5) suggesting these biological processes were stimulated by higher temperatures. Significant linear correlations between temperature and many of these functional parameters (Supplementary Table 15) further suggest that temperature is an important control of many of these parameters. SOD/gAFDM2 was the only functional variable which exhibited a significant negative linear correlation with conductivity after Ln-transformation of both variables (Supplementary Table 15). BfChla (Figure 11B, Supplementary Table 14), on the other hand was higher in spring than in summer-fall, apparently reflecting stimulation by higher light levels. In contrast, none of the denitrification parameters, DeN/gDM (Figure 5B), DeN/gAFDM2 (Figure 5C), DEA/gDM (Figure 12A), and DEA/gAFDM (Figure 12B) exhibited any time or land use * time interactions. Many of the sediment functional indicator values were notably depressed in the Lost samples which may partly reflect the high percentage of barren land use and low RCE scores in the Lost watershed (Supplementary Table 2).

Only SOD/gAFDM2, FDA/gDM, and FDA/gAFDMf of the fourteen functional parameters measured had significant responses to MTR coal mining. The means across all sampling dates were greater in the forested streams than in the MTR-impacted streams for these three functional parameters. No significant responses to MTR coal mining were observed for these three functional parameters on any of the individual sampling dates (Figures 7B, 8A, B). All results presented in this paper are reported in the Supplementary material.

## Discussion

In support of Odum’s hypothesis that functional properties are more resistant to stress induced change than structural properties (Odum, 1985), we found a much higher proportion of structural than functional indicators that differed between MTR-impacted and forested streams. Structural indicator values were higher in the MTR-impacted streams than in the forested streams, which in each case likely reflected source changes or augmentation induced by mining. Further, there were many individual sampling dates in which the MTR value was higher than the forested value for the structural indicators that were significantly higher when all sample dates were combined. In contrast, there were no dates in which the forested values were higher than the MTR values for the functional indicators that showed a significant land use response when all dates were combined. This suggests that the significant structural indicators responded to land use in a more predictable manner than did the significant functional parameters.

High weathering rates resulting from MTR mining cause oxidation of reduced sulfur (e.g., pyrite) to sulfuric acid which then reacts with ^13^C-rich carbonates (δ^13^C ~0 ± 3‰) to produce large quantities of ^13^C-rich DIC which dominate DIC inputs to MTR-impacted streams (Sharma et al., 2013; Vengosh et al., 2013). The δ^13^C-DIC values in the MTR-impacted streams reported here mostly fall within the range of previous measurements (−7–0‰) in streams draining MTR-impacted watersheds (Vengosh et al., 2013; Burke et al., 2014).

On the other hand, soil respiration is likely the dominant source of stream DIC in these forested watersheds (Campeau et al., 2017). Soil CO_2_ levels in forested Appalachian soils were found to exhibit seasonal variation, with near atmospheric levels in winter and up to 10,000–27,000 μatm (25–70 times atmospheric levels) during summer (Rightmire, 1978; Castelle and Galloway, 1990; Jones and Mulholland, 1998). The δ^13^C of DIC entering forested streams is mostly controlled by the δ^13^C of watershed plant materials (−30 to −27‰; Garten and Taylor, 1992), fractionation due to organic matter decomposition (~1.5‰; Acton et al., 2011) and diffusion of CO_2_ from the soil (~4‰; Cerling et al., 1991). After these fractionations, δ^13^C-CO_2_ values between about −26 and −21.5‰ (mean of −23.8‰) from root respiration and organic matter decomposition are expected in the forested soils. The δ^13^C of CO_2_ in forested Virginia soils was also found to vary seasonally, ranging from −9.6‰ in winter to —21.5‰ in summer (Rightmire, 1978). Miller-Tans plots of our data suggest that the mean δ^13^C of DIC contributing to these forested streams is −22.3 ± 1‰ (Figure 3A) and that the δ^13^C of DIC input to the MTR-impacted streams is −2.6 ± 0.8‰ (Figure 3B). These values fall within the range of values predicted above using information from the literature and reinforce the interpretation that MTR coal mining causes a fundamental change in stream DIC source from soil respiration in the forested watersheds to carbonate dissolution in the mined watersheds. Accompanying this source change is a significant change in both DIC concentration and δ^13^C-DIC making these effective indicators of MTR coal mining in these and other MTR-impacted headwater streams.

Several processes such as CO_2_ degassing to the atmosphere, photosynthesis, and in-stream production, both biological and photochemical, can impact stream DIC concentration and δ^13^C-DIC and explain differences between predicted source values and those observed in the stream (Campeau et al., 2017). The generally elevated pCO_2_ values (Supplementary Table 7) and inverse relationship between DIC concentration and δ^13^C-DIC that we observed in the forested streams (Figure 3A) are consistent with degassing as an important controller of δ^13^C-DIC (Doctor et al., 2008). We performed degassing simulations to assess the possible influence of degassing on DIC concentration and δ^13^C. The field data from the forested watersheds largely fits within the two simulation curves (Figure 6A), suggesting that variations in DIC concentration and stable carbon isotopic composition in these watersheds are primarily controlled by evasion of CO_2_ from the streams to the atmosphere (Venkiteswaran et al., 2014; Campeau et al., 2017) and that associated carbon isotopic fractionation is in the 2–3.5‰ range. In contrast, the shape of the plot of DIC vs. δ^13^C-DIC for the MTR-impacted streams bears no resemblance to the degassing simulation curves (Figure 6B), suggesting that degassing is not a significant control of DIC concentration and δ^13^C-DIC in those streams. The elevated pCO_2_ values we observed in the MTR-impacted streams suggests that substantial CO_2_ evasion occurs in those streams; however, acid dissolution of exposed carbonates appears to be a far more important control of DIC concentration and δ^13^C-DIC than degassing in the MTR-impacted streams. Alteration of δ^13^C-DIC by degassing can eventually cause DIC in forested streams to have a δ^13^C within the range of values observed in the MTR-impacted streams, thus rendering δ^13^C-DIC an ineffective indicator of mining influence at advanced stages of degassing. The forested stream samples at an advanced stage of degassing had much lower DIC concentrations than any observed in the MTR-impacted streams, however, so DIC concentration would still be an effective indicator of mining influence (Figures 6A, B).

Groundwater/soil water has been shown to account for about 60% of yearly stream DOC inputs in several southern Appalachian forested watersheds (Meyer and Tate, 1983; Qualls et al., 2002). Instream DOC production from organic matter stored in the stream channel accounts for about 30% of yearly DOC inputs (Meyer et al., 1998). Stream DOC concentrations observed in the present study are very similar to those reported for southern Appalachian mixed hardwood forested watersheds with similar soils and vegetation (Meyer and Tate, 1983; Qualls et al., 2002), suggesting a similarity in stream DOC controls. The δ^13^C of DOC in soil water is generally within ± 2‰ of the δ^13^C of the associated soil organic matter (Amiotte-suchet et al., 2007). Assuming the δ^13^C of soil organic matter in these forested watershed soils ranges from −27 to −25.5‰ (Acton et al., 2011), we would expect soil water δ^13^C-DOC to range from −29 to −23.5‰. This easily encompasses the range of forested stream δ^13^C-DOC values we observed (Figure 4B, Supplementary Table 6) and further suggests the importance of soil water as a source of DOC to these streams. Forested stream δ^13^C-DOC values observed in the present study are also consistent with published values of instream and throughfall sources, falling within the range of C_3_ vegetation δ^13^C, −30 to −27‰ (Garten and Taylor, 1992; Garten et al., 2000).

Two lines of evidence suggest that GOM might be contributing appreciable DOC to the MTR-impacted streams. Acton et al. (2011) reported a mean δ^13^C of −24.7‰ for GOM input to the surface soils of Central Appalachian (Kentucky) watersheds impacted by MTR mining. This δ^13^C of the Kentucky GOM is near the middle of the δ^13^C range of coals (−27 to −22‰; Whiticar, 1996) and nearly identical to the average value (−24.8‰) for Mississippian and Pennsylvanian Type III kerogen reported by Kotarba et al. (2014). Assuming a δ^13^C-GOM of −24.7‰ and that little carbon isotopic fractionation is associated with the solubilization of GOM to DOC, the higher stream δ^13^C-DOC values observed in the MTR-impacted streams (Table 1) suggest the input of geogenic organic carbon (GOC) to the stream DOC pool. Using a δ^13^C-GOC value of −24.7‰, a range of assumed δ^13^C-DOC derived from the present forested stream data, and a simple isotopic mass balance model we calculate that GOC would have to comprise from 15 to 32% of the carbon input to the MTR-impacted streams to account for their higher δ^13^C-DOC values (Supplementary Table 16).

Meyer and Tate (1983) reported that DOC exports from a watershed that had been clear-cut 2 years previously were only about 70% of DOC exports from an undisturbed southern Appalachian forested watershed, due presumably to reduced litter inputs to clear-cut watershed soils. Because MTR mining includes clear-cutting, lower stream DOC concentrations might be expected due to reduced inputs from watershed terrestrial vegetation. Using our discharge and DOC concentration measurements, we calculate that, on average, ~2.7 and 3.2 kg/ha of DOC were transported from the forested and MTR-impacted watersheds, respectively, over the 220-day period for which these measurements are available (Supplementary Table 17). Assuming a 70% reduction of DOC exports from the watershed due to the clear-cut, we calculate that some other source is needed to provide 41% of the DOC (1.3 kg/ha) exported in the MTR-impacted streams (Supplementary Table 17). Along a 14-year reclamation chronosequence of southern Appalachian MTR-impacted soils, GOC dominated the soil organic carbon pool in the upper 50 cm and accounted for 45–99% of the total carbon (Acton et al., 2011), which we calculate amounts to 75–281 Mg GOC/ha. Laboratory extractions with water ranging from <1 day to a week found that about 0.003–1% of the carbon was leached from coal into solution (Fendinger et al., 1989; Orem et al., 1999). The above leaching and carbon pool estimates suggest that about 2–2,810 kg C/ha from leaching of GOC could be available from the upper 50 cm of the soil to potentially make up for the presumed vegetation carbon shortfall in the MTR-impacted watersheds for 1–1,400 years. Leaching of GOM deeper in the valley fills, which typically range from 10 to >100 m in depth (Ross et al., 2016), might provide additional DOC inputs to MTR-impacted streams. These two approaches, isotope mass balance and DOC transport calculations, suggest that GOC could be contributing up to 30–40% of the DOC in the MTR-impacted streams. In support of our contention that GOM plays an important role in the stream C cycle, Fox (2009) estimated that GOM accounted for ~40% of the sediment organic matter load transported by Kentucky streams impacted by MTR mining. Further, transport of GOM particles from MTR-impacted watersheds may contribute to the greater AFDM2 observed in MTR-impacted streams compared to forested streams (Table 1). Slower breakdown of leaf litter in MTR-impacted streams compared to forested streams (Maxwell, 2009; Fritz et al., 2010) would also favor higher AFDM2 levels in mining-impacted streams.

Atmospheric deposition is the dominant, and usually the only, source of reactive N to remote Northeastern forests, which generally leads to relatively low stream nitrate concentrations (Driscoll et al., 2003). Sources of N that likely contribute to the greatly elevated nitrate levels in MTR-impacted streams include N-rich explosives used during the blasting phase, fertilizer used during reclamation, atmospheric deposition in conjunction with reduced plant uptake due to the forest removal stage of MTR, and release of N by the enhanced rock weathering associated with MTR coal mining (Brooks et al., 2019). Although nitrate concentrations are often highly elevated in MTR-impacted streams compared to forested streams, they tend to decline with time since mining to values closer to those of forested streams (Brooks et al., 2019), thus reducing the effectiveness of nitrate concentration as an indicator of MTR mining. Stable N isotope ratios of organisms and organic matter pools have also suggested an alteration in N source or processing as a result of MTR mining (Daniel et al., 2015).

The lack of response to MTR coal mining of most of the functional indicators measured here may be due to the largely intact riparian zones and channel storage (Wondzell and Ward, 2022) which may contribute enough leaf litter to maintain a significant detritus base in the streams, and thus enhance functional stability (Odum, 1969). It was recently reported that in streams with an intact riparian zone, structural, and functional indicators were little affected by forestry disturbance elsewhere in the catchment (Truchy et al., 2022). Our observations are in accord with a previous study in which functional genes associated with microbial metabolism of carbon, nitrogen, sulfur, and selenium were used to evaluate the impact of MTR-mining in a nearby West Virginia watershed (Bier et al., 2020). Less than 10% of the functional genes measured were impacted, some positively due to resource subsidies associated with MTR-mining (e.g., nitrate and sulfate) and some negatively due to carbon limitation and increases in conductivity and metals (Bier et al., 2020).

The counterbalancing of resource subsidies with stressors associated with MTR coal mining (Bier et al., 2020) could also contribute to the unresponsiveness of the functional indicators measured here. On the one hand, sulfate (Pond et al., 2008; Fritz et al., 2010; Ross et al., 2018) and nitrate (Table 1, Figure 5A, Brooks et al., 2019) concentrations are often much higher (7–140 times for sulfate and up to ten times for nitrate) in MTR-impacted streams than in forested streams and may stimulate certain microbial processes. For example, DHA was shown to increase with addition of the electron acceptor sulfate to estuarine sediments (Ho and Liu, 2010) and denitrification rate is positively related to nitrate concentration when an adequate carbon source is available (Seitzinger et al., 2006).

On the other hand, elevated conductivity, which is commonly observed in MTR-impacted streams (Lindberg et al., 2011; Griffith et al., 2012), can depress ecosystem functions. In the present study, conductivity ranged from 49 to 2,513 μS/cm (Supplementary Table 4) and the mean of the MTR-impacted streams (1630 μS/cm) was over 30 times greater than the forested streams’ mean of 52 μS/cm (Johnson et al., 2013). Denitrification rate in saline wetland soils was negatively related to conductivity (negative exponential function) across a conductivity gradient of 2,000–13,000 μS/cm (Yu et al., 2012). Nitrate reductase, a key enzyme in the denitrification process, was negatively correlated with conductivity across a gradient of 457–1,313 μS/cm in stream and river sediments impacted by wastewater treatment plant effluents (Unda-Calvo et al., 2019). Several indices of microbial activity, including FDA and the activities of the extracellular enzymes β-glucosidase, alkaline phosphatase and arylsulfatase were negatively related (negative exponential function) to electrical conductivity over a range of 450–24,000 μS/cm in sugarcane soils (Rietz and Haynes, 2003). DHA, soil respiration, and the activities of several hydrolases were negatively related to electrical conductivity over a range of 140–2,860 μS/cm in arid Mediterranean soils (García et al., 1994). Microorganism-mediated leaf litter breakdown was inversely correlated both to dryland-induced salinity (50–11,000 μS/cm) and coal mining- induced salinity (100–2,400 μS/cm) in stream sediments (Sauer et al., 2016). Metals, which are also often elevated in conjunction with conductivity in MTR-impacted streams (Fritz et al., 2010; Bier et al., 2020), can also depress microbial activity indicators measured here (Hill et al., 1997; Baeseman et al., 2006; Jaiswal and Pandey, 2018).

The complex and heterogeneous chemical composition and overall physical inaccessibility of coal render it difficult to degrade although there are numerous reports of limited biodegradability. Biodegradation of coal by bacteria or fungi includes solubilization, depolymerization, use as a substrate, and methane production (Saha and Sarkar, 2019). Only 1 to 10% by weight of coal was biodegraded during laboratory incubations using various microbial groups and lasting from 14 to 60 days (Laborda et al., 1997; Machnikowska et al., 2002; Fallgren et al., 2013). These results suggest that geogenic organic matter is recalcitrant and, along with elevated conductivity and metals, could also counterbalance the resource subsidies and/or depress or limit microbial process rates if it contributes significantly to the sediment organic matter or DOC pool, which our measurements and calculations suggest it does.

Of the fourteen functional indicators measured, only three—FDA/gDM, FDA/gAFDMf, and SOD/gAFDM2—were detectably different between forested and MTR-impacted streams. The forested stream values were higher in all three cases. The FDA assay provides an estimate of total microbial hydrolytic enzyme activity by bacteria and fungi (Adam and Duncan, 2001) and hydrolytic enzymes are of major importance in leaf litter degradation (Krishna and Mohan, 2017). Leaf breakdown is more rapid in these West Virginian (Maxwell, 2009) and Kentucky (Fritz et al., 2010) forested streams than in corresponding MTR-impacted streams. Leaf litter breakdown in streams is largely mediated by detritivores, fungi, and bacteria (Marks, 2019). Leaf pack shredder density and diversity were higher in these forested streams than in the corresponding MTR-impacted streams, but shredder density was not related to leaf breakdown rate (Maxwell, 2009). Further, fungal biomass accumulation in leaf packs did not differ between these forested and MTR-impacted streams (Maxwell, 2009). It thus appears that shredder density and fungal biomass differences were not the primary drivers of the observed differences in leaf litter breakdown and FDA between these forested and MTR-impacted streams. Rather, it appears that differences in bacterial activity, and possibly shredder diversity were more responsible for the observed differences in leaf litter breakdown and FDA. The observed difference in SOD/gAFDM2 may be partly due to much higher amounts of geogenic organic matter in the MTR-impacted stream sediments (i.e., division of SOD by a larger number) compared to the forested streams. Expressed on a g DM basis, SOD did not differ between forested and MTR-impacted streams (Table 2, Figure 7A). The MTR vs. forested differences in the FDA and SOD parameters likely reflect that (1) the sediment organic matter in the forested streams is more biologically available than that in the MTR-impacted streams and/or (2) stressors associated with MTR coal mining inhibited microbial process rates more than resource subsidies increased them.

The sediment microbial process rate indicators generally exhibit significant time effects with higher values at the higher temperatures (Supplementary Tables 4, 5) observed during fall (16.4–22.4°C) and summer (13.9–25.4°C) as compared to the winter (3.7–8.5°C) and spring (8.5–12.7°C) values in accord with the known influence of temperature on microbial processes. Partial temperature control of these parameters is further demonstrated by the observed significant linear correlations of several of these parameters with temperature (Supplementary Table 15). Hill et al. (2000) also observed a significant linear correlation between SOD/gAFDM and temperature for first- to third-order streams sampled during spring-summer in the Central Appalachians. The biofilm production parameters are less consistently related to temperature and are likely responding to other factors such as hydrodynamics, carbon and nutrient availability, and light (Battin et al., 2016). There is substantial variation in the land use (forest or barren) and riparian zone condition (RCE scores) of the MTR-impacted streams (Supplementary Table 2), and this may partially account for the relatively higher variability in DIC and nitrate concentrations observed in the MTR-impacted streams compared to the forested streams. The barren land use is particularly high and the RCE scores are very low in the Lost watershed, and this may help explain the much lower functional indicator values observed in Lost compared to the other MTR-impacted streams.

Despite the substantial watershed disturbance and change in physicochemical water quality, and the likely reduction in organic matter bioavailability due to inputs of geogenic carbon associated with MTR coal mining, we did not observe significant impacts on most of the microbial process rate indicators measured in this study. Reasons for the muted response of these functional indicators likely include: (1) the continued detritus-base in the MTR-impacted streams due to the largely intact riparian zones and/or channel storage; (2) the diversity of the microbial community which contains stress-tolerant members able to carry out the functions while exposed to MTR coal mining stressors such as elevated conductivity and metals; and (3) negative impacts from MTR coal mining are counterbalanced by associated resource subsidies such as elevated nitrate and sulfate. The short sampling period and small sample sizes of this study, as well as the high variability of many of our functional parameters and the fact that our forested sites have also been subjected to disturbance (Feio et al., 2010), may have also limited our ability to detect functional indicator responses to MTR mining. The link between MTR mining and the structural indicators appears to be simpler, a change in or augmentation of the source(s) of the C, N, and organic matter indicators. These results largely support Odum’s hypotheses that: (1) a mature detritus base confers functional stability to a system; and (2) functional properties are more resistant to stress induced change than structural properties. The ideal indicator would show treatment differences on all sampling dates; under this criterion δ^13^C-DIC and nitrate concentration were the most effective indicators in the present study. The other structural indicators and all functional indicators exhibited fewer or no treatment differences on the individual sampling dates. These inefficient indicators would need to be sampled more times to potentially detect a treatment difference. Our results illustrate the difficulty of identifying effective functional indicators for evaluating the ecological integrity of stressed stream ecosystems.

## Supplementary Material

Supplement1

Supplement2

## Figures and Tables

**TABLE 1:** Structural indicators mean [concentration], range, and *p*-values from model with lowest AICc.

Indicator	Mean (range) F	Mean (range) M	Land use *p*	Time *p*	Land use * Time *p*
**[DIC]** (mg/L)	**1.63** (0.35–6.95)	**18.05** (2.4–54.5)	*0.03*	*<0.001*	*0.03*
***δ***^13^**C-DIC** (‰)	**−12.0** (−21.6 to −5.3)	**−4.9** (−13.4 to −1.4)	*<0.001*	*<0.001*	*<0.01*
[DOC] (mg/L)	**0.87** (0.40–1.41)	**1.04** (0.81–1.88)	0.09	*0.04*	*<0.01*
***δ***^13^**C-DIC** (‰)	**−27.7** (−28.7 to −27.0)	**−27.0** (−28.1 to −24.2)	*<0.001*	*<0.01*	*0.02*
**[Nitrate]** (μg N/L)	**370** (45–711)	**3,561** (877–6,012)	*<0.001*	0.86	0.76
pCO_2_ (μatm)	**1,052** (402–2,541)	**1,777** (60–6,370)	0.07	*0.02*	0.06
**AFDM2—DEA (‰)**	**1.7** (0.8–2.7)	**3.7** (1.5–10.4)	*<0.001*	0.15	0.34
**AFDM2—SOD (‰)**	**1.9** (1.0–3.0)	**3.5** (1.9–7.0)	*0.02*	0.16	0.75
AFDMf—FDA (‰)	**15.8** (9.5–23.7)	**12.1** (8.6–26.1)	0.13	0.40	0.22
AFDMf—DHA (‰)	**14.3** (2.8–23.9)	**11.5** (6.4–21.1)	0.14	*0.02*	0.39

F, forested; M, mined. Significant p-values in italics. Variables with significant land use differences shown in bold and underlined. See Supplementary Table 5 for more details.

**TABLE 2:** Functional indicator *p*-values from models with the lowest AICc.

Indicator	Mean (range) F	Mean (range) M	Land use *p*	Time *p*	Land use * Time *p*
DEA/gDM (μg N/gDM/d)	**1.5** (0.1–4.8)	**2.1** (<0.1–9.8)	0.13	0.28	0.48
DEA/gAFDM2 (μg N/gAFDM2/d)	**91.3** (6.8–292)	**61.1** (<0.1–223)	0.24	*0.02*	0.38
DeN/gDM (μg N/gDM/d)	**1.0** (0.1–2.7)	**1.9** (<0.1–9.7)	0.24	*0.03*	0.50
DeN/gAFDM2 (μg N/gAFDM2/d)	**70** (4.7–204)	**42** (0.01–128)	0.14	0.07	0.80
SOD/gDM (μg O_2_/gDM/hr)	**1.8** (0–4.0)	**1.7** (0.2–8.8)	0.63	*0.01*	0.06
**SOD/gAFDM2** (mg O_2_/gAFDM2/hr)	**0.09** (0–0.28)	**0.05** (<0.01–0.28)	*0.03*	*0.05*	0.07
**FDA/gDM** (μmol FDA/gDM/d)	**618** (86–2,086)	**191** (3–580)	*0.03*	*<0.001*	0.07
**FDA/gAFDMf** (μmol FDA/gAFDMf/d)	**3,644** (585–11,243)	**1,708** (10–6,119)	*<0.01*	*<0.001*	0.32
DHA/gDM (μmol INTF/gDM/d)	**0.99** (0.23–2.58)	**1.40** (0.33–4.03)	0.08	*0.04*	0.91
DHA/gAFDMf (μmol INTF/gAFDMf/d)	**7.20** (3.84–15.48)	**11.38** (2.24–25.64)	0.11	*0.04*	0.45
BfOD (g O_2_/d/m^2^/d)	**0.02** (<0.01–0.12)	**0.01** (0–0.10)	0.38	*0.05*	0.83
BfFDA (nmol FDA/hr/cm^2^/d)	**6.91** (0.36–29.50)	**8.61** (0.93–27.33)	0.08	*<0.001*	*0.03*
BfDHA (nmol INTF/d/cm^2^/d)	**1.32** (0.31–3.48)	**1.07** (0–4.90)	0.45	*0.01*	0.41
BfChla (mg/m^2^/d)	**0.54** (0.06–1.43)	**0.68** (0.03–2.50)	0.32	*<0.001*	0.07

Significant p-values in italics. Variables with significant land use differences shown in bold and underlined. See Supplementary Table 5 for more details.

## Data Availability

The datasets presented in this study can be found in the Supplementary material and at https://doi.org/10.23719/1528387.
