# Supplementary figures and images for: Mountaintop removal coal mining impacts on structural and functional indicators in Central Appalachian streams
Source: Front Water. Author manuscript; Available in PMC 2024 Jan 19. (PMC10031508; doi:10.3389/frwa.2022.988061)

## Slide 1
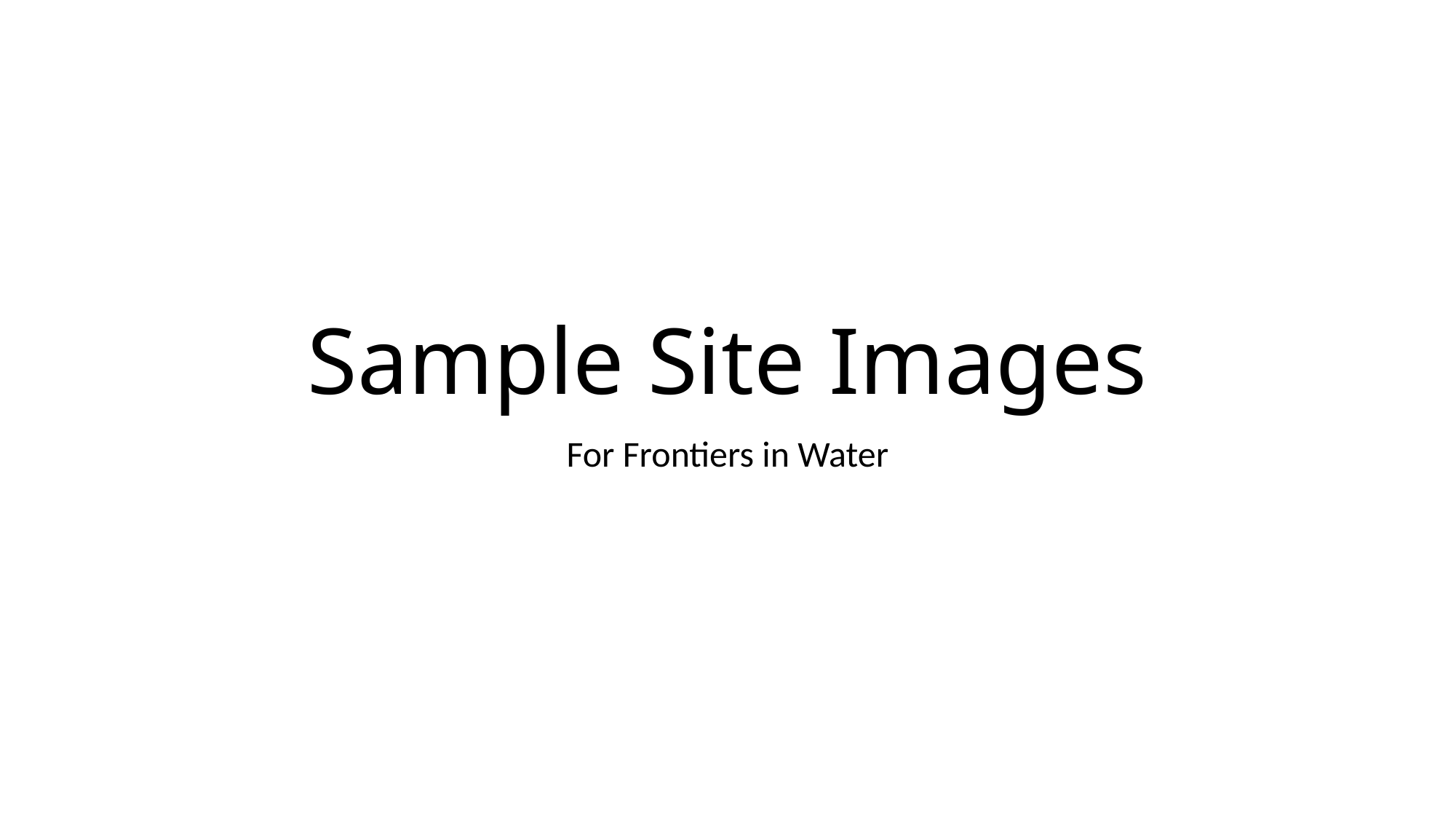

# Sample Site Images
For Frontiers in Water

## Slide 2
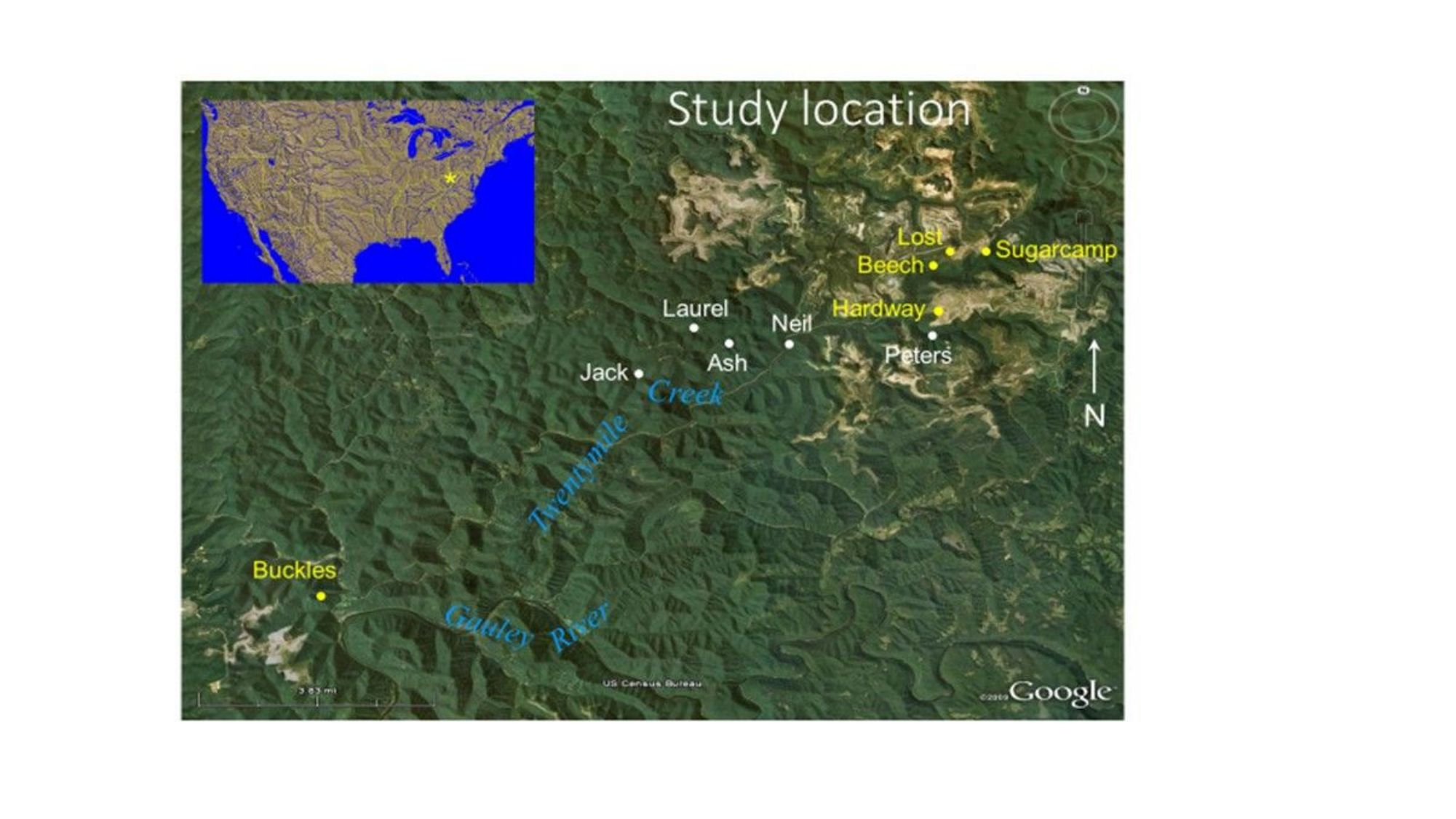

## Slide 3
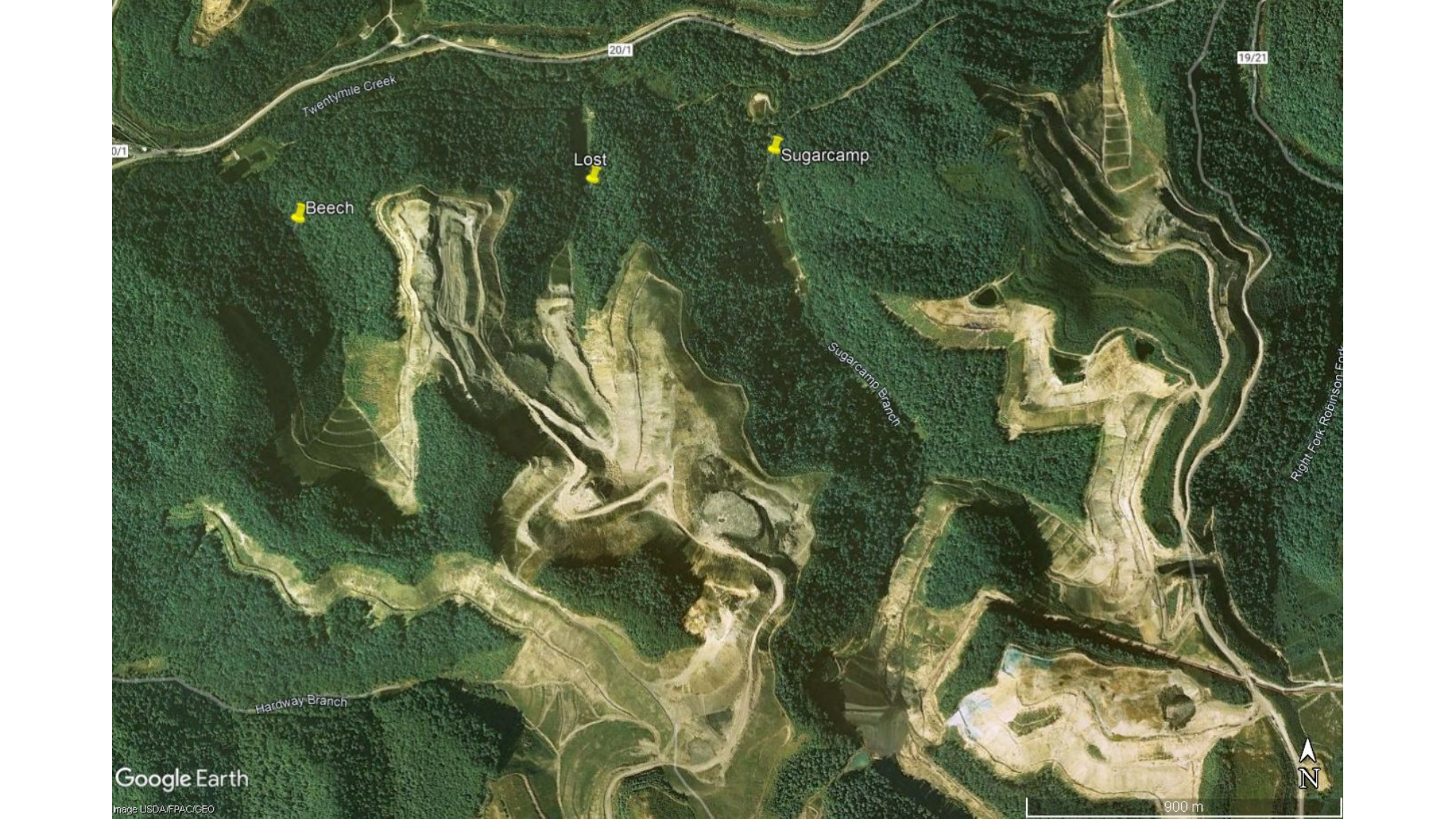

## Slide 4
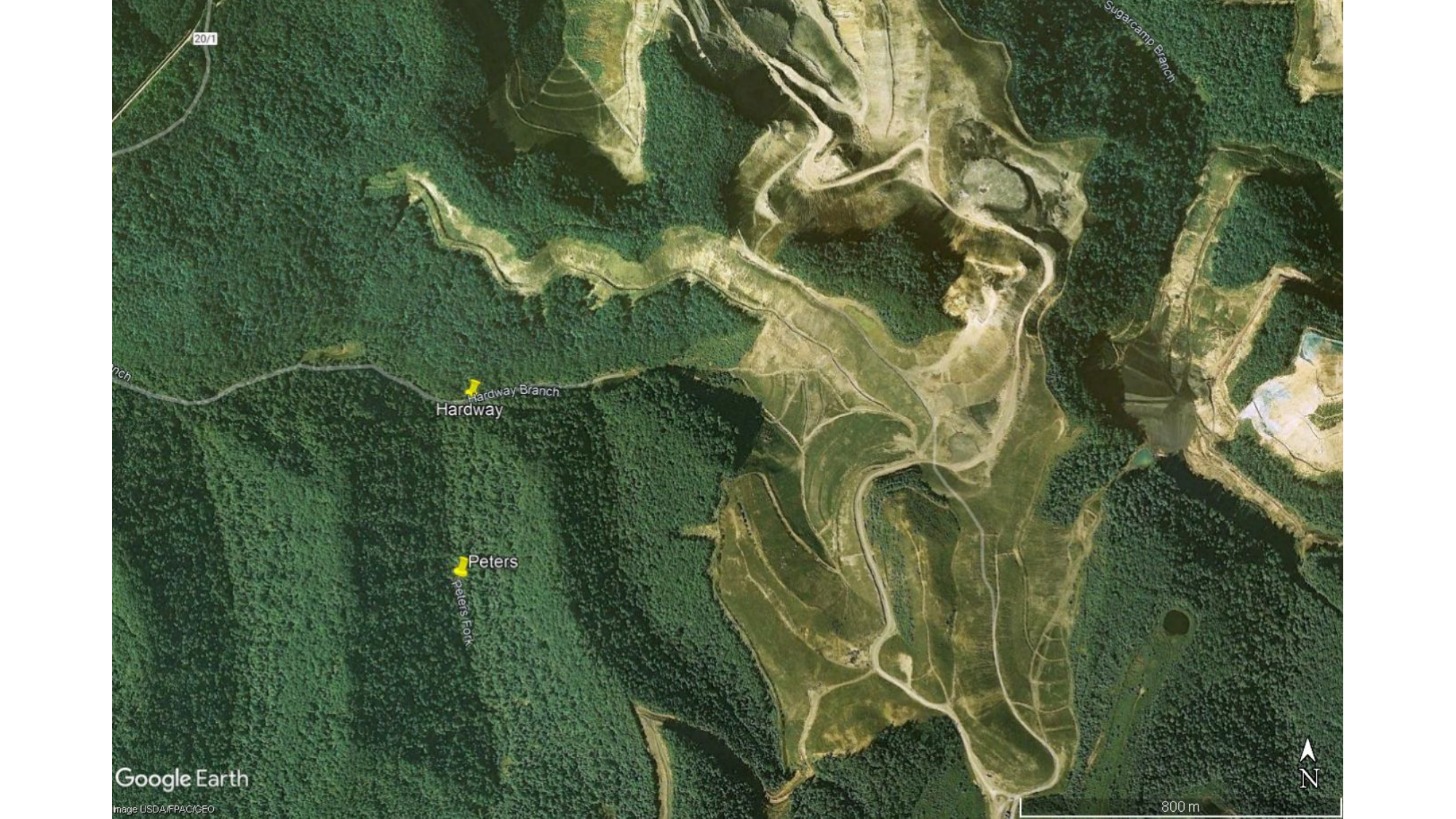

## Slide 5
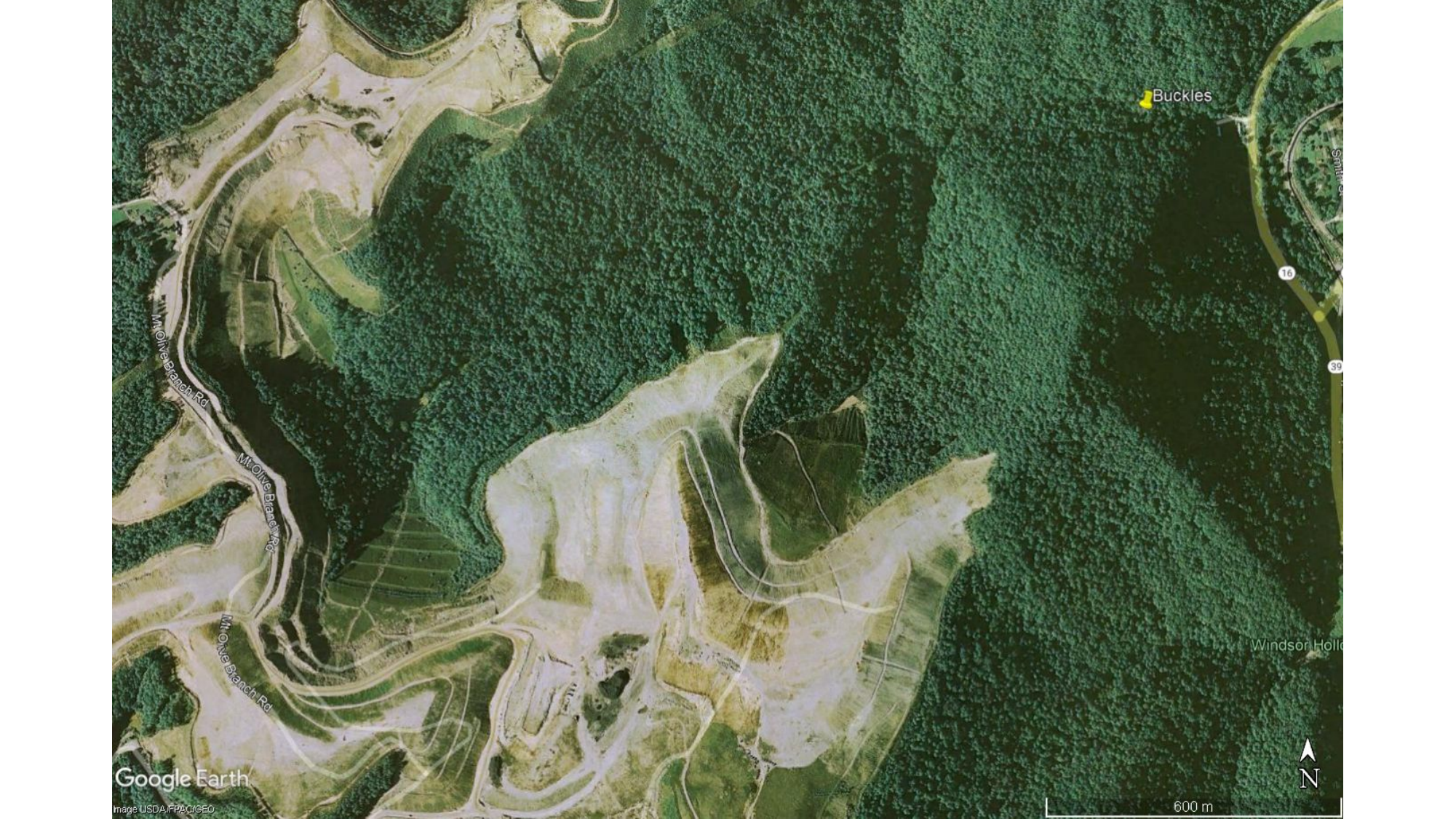

## Slide 6
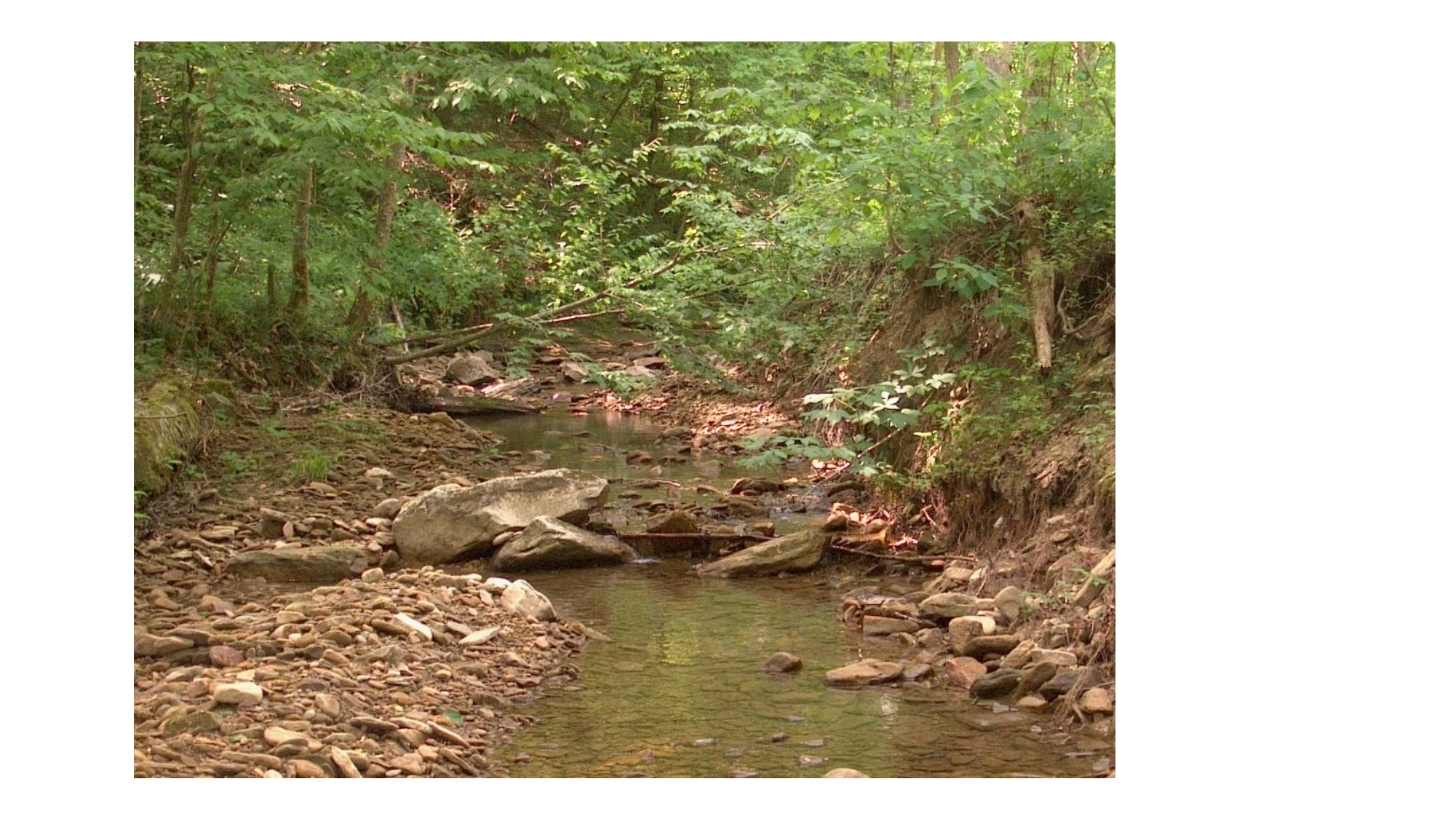

## Slide 7
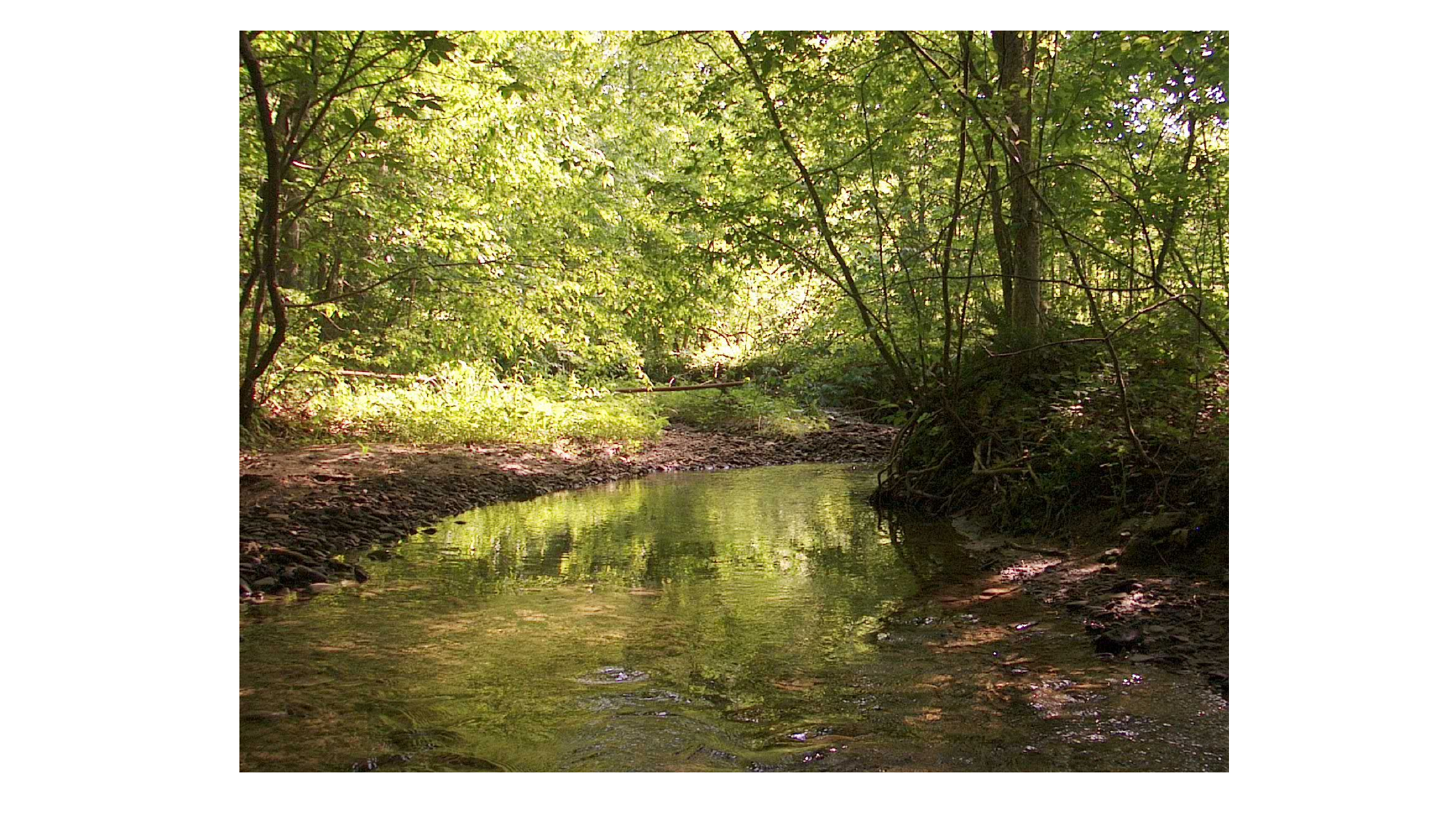

Supplement: Supplement2 [file NIHMS1877571-supplement-Supplement2.pptx]
